# Semi-Automated Cell and Tissue Analyses Reveal Regionally Specific Morphological Alterations of Immune and Neural Cells in a Porcine Middle Cerebral Artery Occlusion Model of Stroke

**DOI:** 10.3389/fncel.2020.600441

**Published:** 2021-01-22

**Authors:** Samantha E. Spellicy, Kelly M. Scheulin, Emily W. Baker, Brian J. Jurgielewicz, Holly A. Kinder, Elizabeth S. Waters, Janet A. Grimes, Steven L. Stice, Franklin D. West

**Affiliations:** ^1^Regenerative Bioscience Center, University of Georgia, Athens, GA, United States; ^2^Medical College of Georgia, University System of Georgia MD/Ph.D. Program, Augusta, GA, United States; ^3^Biomedical and Health Sciences Institute, Neuroscience Program, University of Georgia, Athens, GA, United States; ^4^Department of Animal and Dairy Sciences, University of Georgia, Athens, GA, United States; ^5^Aruna Bio Inc., Athens, GA, United States; ^6^Department of Small Animal Medicine and Surgery, College of Veterinary Medicine, University of Georgia, Athens, GA, United States

**Keywords:** cell morphology, permanent middle cerebral artery occlusion model, porcine, ischemic stroke, high-content imaging

## Abstract

Histopathological analysis of cellular changes in the stroked brain provides critical information pertaining to inflammation, cell death, glial scarring, and other dynamic injury and recovery responses. However, commonly used manual approaches are hindered by limitations in speed, accuracy, bias, and the breadth of morphological information that can be obtained. Here, a semi-automated high-content imaging (HCI) and CellProfiler histological analysis method was developed and used in a Yucatan miniature pig permanent middle cerebral artery occlusion (pMCAO) model of ischemic stroke to overcome these limitations. Evaluation of 19 morphological parameters in IBA1^+^ microglia/macrophages, GFAP^+^ astrocytes, NeuN^+^ neuronal, FactorVIII^+^ vascular endothelial, and DCX^+^ neuroblast cell areas was conducted on porcine brain tissue 4 weeks post pMCAO. Out of 19 morphological parameters assessed in the stroke perilesional and ipsilateral hemisphere regions (38 parameters), a significant change in $\frac{38}{38}$ measured IBA1^+^ parameters, $\frac{34}{38}\;$GFAP^+^ parameters, $\frac{32}{38}$ NeuN^+^ parameters, $\frac{31}{38}$ FactorVIII^+^ parameters, and $\frac{28}{38}$ DCX^+^ parameters were observed in stroked vs. non-stroked animals. Principal component analysis (PCA) and correlation analyses demonstrated that stroke-induced significant and predictable morphological changes that demonstrated strong relationships between IBA1^+^, GFAP^+^, and NeuN^+^ areas. Ultimately, this unbiased, semi-automated HCI and CellProfiler histopathological analysis approach revealed regional and cell specific morphological signatures of immune and neural cells after stroke in a highly translational porcine model. These identified features can provide information of disease pathogenesis and evolution with high resolution, as well as be used in therapeutic screening applications.

## Introduction

Stroke remains a leading cause of death and long-term disability worldwide despite numerous preclinical and clinical trials to develop novel treatments (Virani Salim et al., 2020). The development of new therapeutic targets and more effective treatments hinge on having a more complete understanding of cellular dynamics after stroke to assess changes in key processes such as the immune response and glial scar formation, which may also serve as biomarkers of injury severity and recovery. Modern histological approaches have led to significant breakthroughs in understanding thrombus etiology (Sporns Peter et al., 2017; Heo et al., 2020), patterns of immune cell activation (Savchenko et al., 2016; Rayasam et al., 2018), and other important pathological and recovery cellular changes. However, these standard approaches are limited by the number of cellular features that can be assessed, accompanied by decreased spatial information, slow data processing speeds, and subjectivity. Recent advances in imaging and processing power have led to the development of high-content image (HCI) analysis that enables high-throughput assessment of multiple single cell features, reduces time of analysis, and decreases subjectivity (Chen et al., 2012; Riordan et al., 2015). Assessment of micro-scale cellular morphological changes, garnered through HCI analysis of stroked tissues, is likely to result in more predictive studies of neural injury and recovery responses- a critical need in the stroke field (Kaiser and West, 2020).

HCI individual cell analysis has been utilized to better understand the dynamic changes in cellular morphological features in the injured brain (Morrison and Filosa, 2013; Heindl et al., 2018). Recent studies have identified distinct morphological changes in microglia in basic shape descriptions, such as surface area and perimeter, skeleton properties of ramified cells, and graph theory parameters in the peri-infarct region after ischemic stroke in rodent models. Additionally, automated analysis has resulted in substantial decreases in time to result acquisition (5 min vs. 7 h) relative to manual approaches, with no compromise of result integrity (Heindl et al., 2018). However, the limited number of *in vivo* HCI stroke studies have focused on either a well-demarcated perilesional region or a specific brain structure (Brown Craig et al., 2008; Heindl et al., 2018), rather than global ipsilateral hemisphere changes. While region-specific differences in cellular morphology have been identified, such as in the case of astrocyte morphology (Mestriner et al., 2015), analysis of the peri-infarct region as well as the ipsilateral hemisphere should be conducted to determine local and global alterations in cellular morphology. For example, significant differences in cellular morphology of microglia within the infarct and a few millimeters from the infarct region have been previously observed, demonstrating the existence of transitions in morphological populations (Heindl et al., 2018). Examining differences in inflammatory cell morphology changes induced by ischemia, edema, intracerebral hemorrhage, or even in response to therapy, may provide more descriptive information about the pervasiveness of injury throughout the affected issue than solely considering the direct lesioned area alone. To date, most HCI neural analysis studies have focused primarily on individual cell assessments of only a few hundred of the billions of brain cells with an emphasis typically on one specific cell type such as microglia/macrophages (Thored et al., 2009; Heindl et al., 2018), neurons (Gonzalez and Kolb, 2003), or astrocytes (Wilhelmsson et al., 2006; Mestriner et al., 2015). While these studies provide significant detail into the changes of single cell-type features, they neglect other key cell types and relationships between these cells that contribute to normal and injured brain activity. For example, morphological changes of IBA1^+^ microglia/macrophages and GFAP^+^ astrocytes following activation has been documented in many studies of neuroinflammation (Karperien et al., 2013; Hovens et al., 2014) and stroke specifically (Heindl et al., 2018). Activation of IBA1^+^ microglia/macrophages is associated with a transition from ramified, extended cells with a large perimeter to area ratio, to cells that are more ameboid, compact, and have a smaller perimeter phenotype (Avignone et al., 2015; Davis et al., 2017). Additionally, activation of GFAP^+^ astrocytes proximal to the lesion site exhibit a stellate, hypertrophied appearance with processes extending toward the ischemic core at acute timepoints during glial scar formation (Ding, 2014). However, the relationship between the morphological changes in each of these cell types to each other has yet to be quantitatively characterized. The improved understanding of these relationships between cell types is likely to be important in investigating novel cell interaction in the stroke environment and discovering new therapeutic targets and mechanisms.

Utilizing animal models with similar neural cellular composition and organization as humans in preclinical stroke research is becoming increasingly important to improve the potential of successful clinical translation (Kaiser and West, 2020). In this study, the porcine brain is utilized for HCI cellular histological analysis following pMCAO due to similar white matter content, size, and hierarchical organization to the human brain (Platt et al., 2014). White matter is exceedingly vulnerable to ischemic damage relative to gray matter due to the lack of sufficient collateral circulation, excitotoxicity susceptibility, and destruction of damaged oligodendrocytes by microglia and infiltrating macrophages (Moxon-Emre and Schlichter, 2010). Therefore, for translational purposes, it is imperative to quantify morphological changes of inflammatory and neural cells in large animal brains that show greater similarity to the human stroke condition.

To comprehensively investigate quantitative cellular morphological changes after stroke, a custom, semi-automated HCI and CellProfiler analysis pipeline (Jones et al., 2009) was used to capture 19 morphological features of 5 different cell types in a pMCAO porcine model of ischemic stroke. While the methodology and analysis pipeline presented here are generalizable to small animal stroke models, it is especially useful in the quantification of cells and associated aspects in large animal models that have sizable brain mass, which historically has made detailed quantitative analysis of discrete segments challenging. This study demonstrated significant differences in the morphology of microglia/macrophages, astrocytes, neurons, neuronal precursors, and vasculature cells in the stroked porcine brain. Additionally, significant correlations were observed between morphological changes in these cell types following stroke, suggesting that this HCI histological analysis approach can lead to new information on the dynamic interplay between cytoarchitectural and inflammatory cell types in the post-stroke brain.

## Materials and Methods

### Study Design

All work involving the use of animals or animal tissue in this study was performed in accordance with the National Institutes of Health (NIH) Guidelines for Care and Use of Laboratory Animals and was reviewed and approved by the University of Georgia (UGA) Institutional Animal Care and Use Committee (IACUC; Animal Use Protocol (AUP) Number A2018 01-029-Y1-A5). Pigs were acquired from Lonestar Laboratory Swine (Sioux Center, IA) and individually housed in a Public Health Service (PHS) and Association for Assessment and Accreditation of Laboratory Animal Care (AAALAC) approved facility. Animal rooms were maintained at temperature of approximately 27°C with a 12-h light/dark cycle. Pigs were fed standard UGA grower 1 diet with daily enrichment through toys and human contact. Predefined exclusion criteria included instances that would prevent the animal from reaching the 4-week timepoint such as death or euthanasia due to severe self- inflicted injuries, inability to thermoregulate, uncontrolled seizure activity, respiratory distress, and/or confounding infections at the incision. No pigs reached the aforementioned exclusion criteria, and therefore all animals were included in the analysis.

### Porcine Ischemic Stroke Surgery

In this single-blinded study, sexually mature [>1 year] male and ovariectomized (OVX) female Yucatan miniature pigs weighing between 68 and 98 kg were randomly assigned to either non-stroke (NS, *n* = 3 females, *n* = 2 males) or stroke (S, *n* = 3 females, *n* = 4 males) groups. Due to the global effect of the ischemic injury on the entire brain, normal healthy animals were selected to be used as the control group rather than the contralateral hemisphere. The day prior to surgery, pigs were administered antibiotics (Ceftiofur crystalline free acid; 5 mg/ kg intramuscular (IM); Zoetis) to prevent infection as required by institutional guidelines. A right sided pMCAO procedure was performed on all stroked animals by a trained veterinary neurosurgeon as described in Platt et al. (2014). Briefly, pre-induction analgesia and sedation was achieved using xylazine (4 mg/kg IM; Vet-One), midazolam (0.3 mg/kg IM; Heritage), and methadone (0.2 mg/kg IM; Henry Schein Animal Health). Anesthesia was induced with propofol (to effect; intravenous (IV); Zoetis) and prophylactic lidocaine (1.0 mL 2% lidocaine; VetOne) was applied to the laryngeal folds to facilitate intubation. Anesthesia was maintained with isoflurane (1.0–2.0%; Abbot Laboratories) in oxygen. Pigs were determined to be at an adequate anesthetic depth when animals displayed complete muscle relaxation and no longer responded to a toe pinch or ear stimulation per AUP. For anesthetic depth monitoring during anesthesia events, heart rate and blood pressure were monitored using a stethoscope and/or Doppler Probe every 5 min, respiration rate was regulated using a ventilator every 5 min, and temperature was monitored using a thermometer every 15 min. Following a frontotemporal craniectomy with orbital rim ostectomy and zygomatic arch resection, the temporal fascia and muscle were elevated, and the local dura mater was exposed. The middle cerebral artery (MCA) and collaterals were then identified just distal to the Circle of Willis. Permanent occlusion of the distal MCA and associated branches was achieved by bipolar electrocauterization resulting in territorial ischemia. The exposed brain was then covered with a sterile oxidized cellulose hemostatic agent (VetSpon). Following occlusion, the temporalis muscle and epidermis were routinely re-apposed. Once the incision was closed, anesthesia was discontinued, pigs were returned to their pens, and extubated. Temperature, respiration, and heart rate were monitored every 15 min until vitals returned to a normal range. Additionally, vitals were observed every 4 h for 24 h and then pigs received health checks twice daily until completion of the study. To control for postoperative pain, acute inflammation, and fever management, banamine (2.2 mg/kg IM or IV; Merck) was administered every 12 h for 24 h and every 24 h for 3 days following surgery and methadone (0.2 mg/kg IM or IV; Henry Schein Animal Health) was administered every 6 h for 24 h following surgery.

### Porcine Magnetic Resonance Imaging

Pigs underwent magnetic resonance imaging (MRI) at 1-day post-pMCAO to confirm ischemic stroke and determine brain infarct volume and patterning. MRI was acquired on a GE Signa HDx 3.0 Tesla scanner using an 8-channel large torso coil with the pig positioned in supine recumbency under general anesthesia described during stroke surgery. Fast Spin Echo (FSE) T2-Weighted (T2W) and Spin Echo (SE) Diffusion Weighted Imaging (DWI) were acquired in the multiplanar MRI protocol. Apparent diffusion coefficient (ADC) maps were generated from DWI sequences. Using OsiriX software (Version 10.0.5, Pixmeo SARL, Bernex, Switzerland), hemisphere volumes were calculated utilizing T2W sequences and ischemic lesion volumes were calculated utilizing ADC maps, as previously described (Gerriets et al., 2004). To control for edema in the brain due to the stroke, DWI corrected lesion volumes were calculated according to the following formula modified from Loubinoux et al. (1997) and Webb et al. (2018). LV^c^ = HV_c_+HV_i_-(HV_c_+HV_i_-LV^u^)^*^($\frac{HVc+HVi}{2HVc}$) where lesion volume corrected (LV^c^), lesion volume uncorrected (LV^u^), contralateral hemisphere volume (HVc), and ipsilateral hemisphere volume (HVi). A representative MRI image of the ischemic lesion patterning is demonstrated in Supplementary Figure 9A.

### Porcine Histology

Four weeks after pMCAO, NS and S pigs were euthanized by IV pentobarbital (1 mL/4.5 kg) injection. Brains from both groups were removed and immersed in 10% buffered formalin (Millipore Sigma). Next, brains were sectioned coronally using a pig brain slicer (Zivic Instruments, Pittsburgh, PA). Right (ipsilateral to pMCAO) hemisphere sections were formalin-fixed, embedded in paraffin, and sectioned for immunohistochemistry (Leica RM2255, Germany). Following an enzyme block in 3% H_2_O_2_ for 5 min and Power Block (BioGenex) for 5 min, 4 μM thick sections were incubated with primary antibodies for 1 h on a Biocare Medical Nemesis 7200 Autostainer. Primary antibodies used were GFAP (mouse 1:4,000, Biogenex MU020-UC, Clone GA-5), IBA1 (rabbit 1:8,000, Wako, 019-19741), NeuN (guinea pig 1:3,000, Millipore Sigma, ABN90), FactorVIII (rabbit, Cell Marque, 250A-18), and DCX (rabbit, 1:4,000, Abcam, ab18723). Secondary antibodies used were Biotinylated goat anti-rabbit 1:100, Vector Labs, BA-1000 for IBA1, Biotinylated horse anti-mouse 1:100, Vector Labs, BA-2001 for GFAP, Biotinylated goat anti-guinea pig 1:100, Vector Labs, BA-7000 for NeuN, Biotinylated goat anti-rabbit 1:100, Vector Labs, BA-1000 for FactorVIII, and Biotinylated goat anti-rabbit 1:100, Vector Labs, BA-1000 for DCX. The substrate is DAB + Chromogen (12 min) from Biocare and all were counterstained with hematoxylin.

### Morphological Profiling

**Figure 1:**
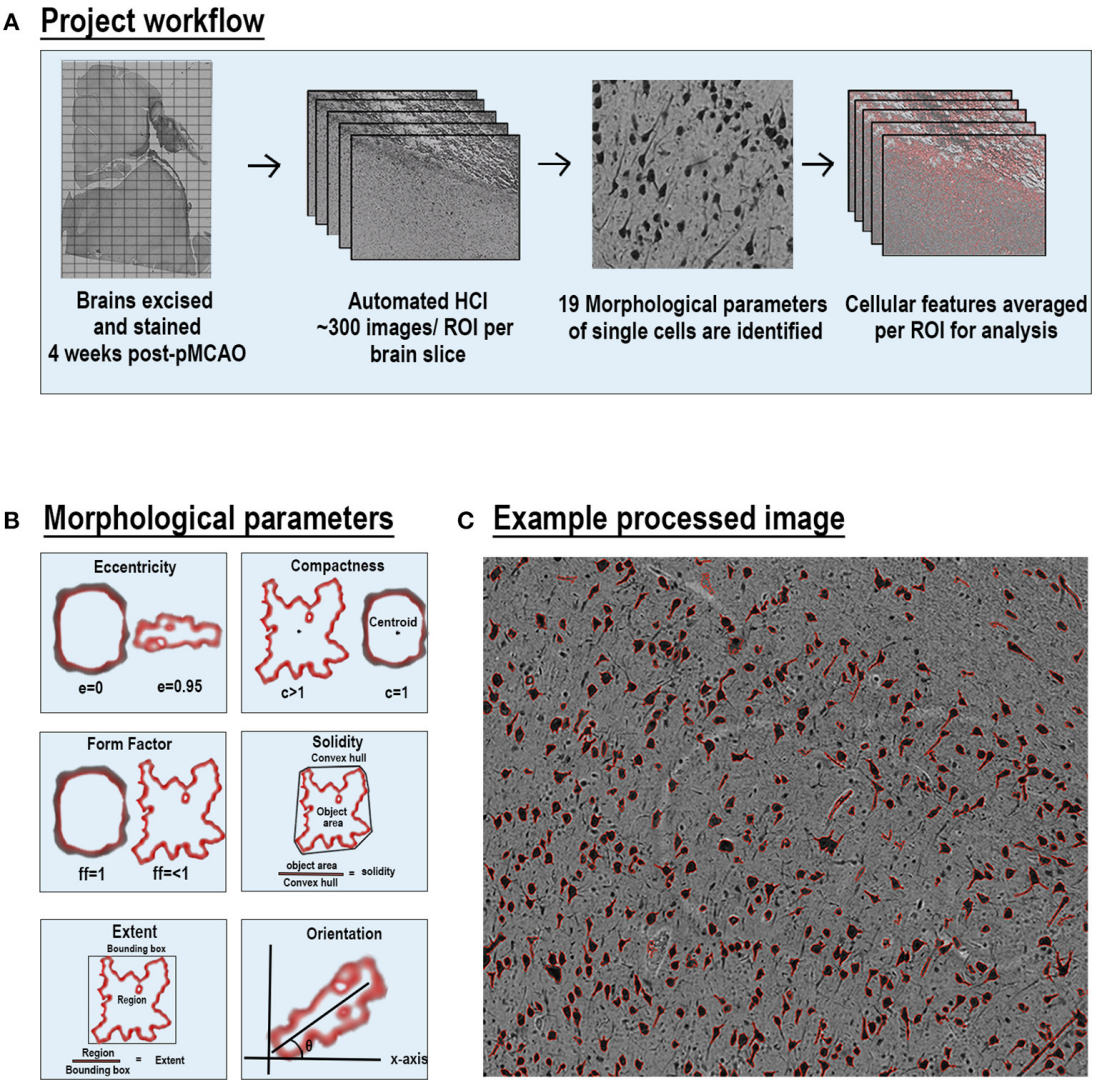
Automated high-content image processing pipeline and morphological parameters. Diagram of multiparametric HCI processing pipeline of cellular features from S and NS animals **(A)**. Explanation of 6 of the 19 morphological parameters measured for each cell per ROI **(B)**. Representative NeuN stained ROI after processing with outlines (red) of each positve cell area **(C)**.

Each stained ipsilateral hemisphere slide was imaged using a Cytation 5 Imaging Multi-mode reader (BioTek, Vermont) in a 12 × 19–30 grid. This acquisition resulted in between 209 and 330 images per section, each representing a separate region of interest (ROI) per ipsilateral section. Individual images from each ipsilateral hemisphere were analyzed through a custom CellProfiler pipeline for individual stains (Figure 1A). The pipeline was run on a Dell 5820 Tower Workstation using a Windows 10 Pro operating system and powered by a Xenon W-2133 3.6 Gigahertz processor with 32 gigabytes (GB) of memory and 256 GB of solid-state drive (SSD). CellProfiler detailed 19 parameters including area occupied, count, perimeter, shape area, compactness, eccentricity, Euler number, extent, form factor, major axis length, max ferret diameter, area shape maximum radius, area shape mean radius, area shape median radius, min ferret diameter, area shape minor axis length, orientation, area shape perimeter, and solidity per positive area or cell. All units represented are either in pixels or are unitless. Definitions and equations for each parameter are defined in Supplementary Table 1 and examples are provided in Figures 1B,C.

The values of each parameters for each positive area were averaged for a given ROI. ROI averages were used for statistical comparisons between NS and S groups. All ROIs per section were used for ipsilateral hemispheric section comparisons (~300 ROIs per animal), and only ROIs from the perilesional area were used for perilesional comparisons (~60 ROIs per animal). In NS animals, an equal number of ROIs from the corresponding neuroanatomical locations of S animals were used for perilesional histological analysis. Principal component analysis (PCA) was conducted including all of the morphological features previously described. Each dot in the plot represents 1 ROI from 1 animal.

Whole processed and stitched images from each animal for IBA1 (Supplementary Figure 4), GFAP (Supplementary Figure 5), NeuN (Supplementary Figure 6), FactorVIII (Supplementary Figure 7), and DCX (Supplementary Figure 8) are displayed in Supplementary Materials.

### Statistical Analysis

Morphological parameters from each positive area were recorded and quantified. Per cell or per area analysis was then averaged in each ROI captured. Average of each ROI (ranging from 100s to 10,000s, depending on stain and location) was compared. A normality test was conducted for each parameter and a Mann-Whitney test was utilized for non-parametric data (GraphPad Prism 8). For heatmap generation and correlation analysis, all ROIs were averaged to a per-pig level to allow for comparison of cell type-specific morphological variation within each pig. All 10 parameters of each treatment group were compared utilizing PCA and Welch's unpaired *t*-test to determine significant differences between principal components of NS and S animals. Simple linear regression analysis was conducted between each pig's lesion volume and area of each stain per whole section (GraphPad Prism 8).

## Results

### IBA1^+^ Areas Transitioned to a More Ameboid and Swollen Morphology After Stroke

**Figure 2:**
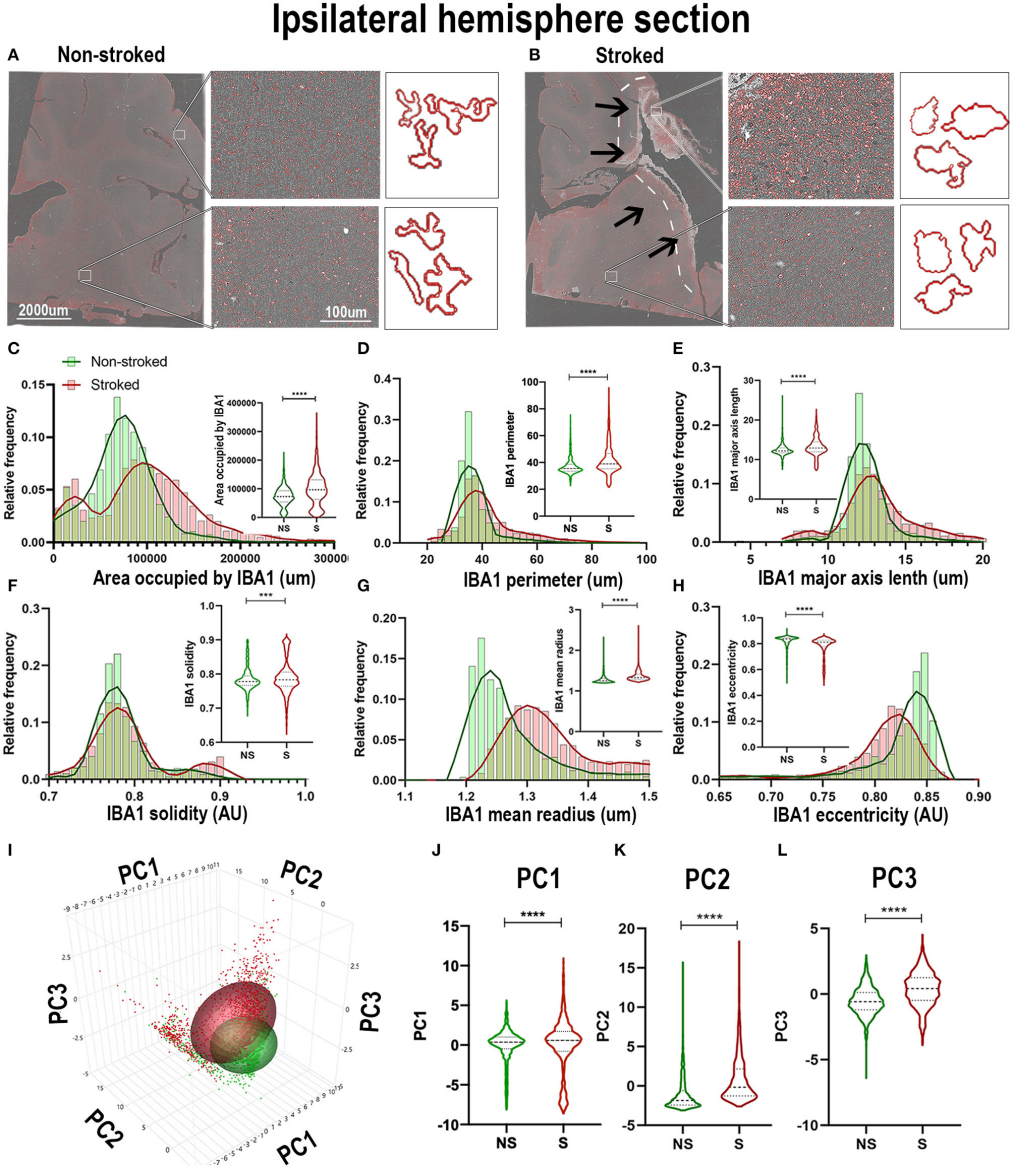
IBA1^+^ areas transitioned to a more ameboid and swollen morphology after stroke in the ipsilateral hemisphere. Visual differences in density and morphological changes of IBA1^+^ areas were observed between NS and S animals **(A,B)**. Arrows indicate location of stroke lesion **(B)**. Significant (*p* < 0.0001) increases in IBA1+ area occupied **(C)**, perimeter **(D)**, major axis length **(E)**, solidity (**F**; *p* = 0.0008), and mean radius **(G)** were observed in hemisphere section of S (*n* = 1,860 ROIs) compared NS (*n* = 1,362 ROIs). There was a significant (*p* < 0.0001) decrease observed in eccentricity **(H)** of IBA1^+^ ROIs in hemisphere section analysis in S animals compared to NS animals. PCA **(I)** analysis showed significant (*p* < 0.0001) increases in PC1 - PC3 **(I–L)** in S compared to NS animals. **p < 0.001 and ****p < 0.0001.

Qualitatively, positive area outlines generated through CellProfiler analysis revealed a visual difference in the size of IBA1^+^ areas throughout the ipsilateral sections and perilesional regions in S animals compared to NS animals (Figures 2A,B). In addition, morphological changes of individual IBA1^+^ cells in NS animals presented a more extended, ramified appearance compared to S animals, which presented a more rounded, ameboid appearance. Quantification of morphological parameters of IBA1^+^ area in the ipsilateral hemispheric section revealed significant differences between S (n=1,860 ROIs) and NS (n=1,362 ROIs) animals. A significant increase in IBA1^+^ area occupied (Figure 2C; Median NS = 72,843px, S = 96,963px, *p* < 0.0001), IBA1^+^ area shape perimeter (Figure 2D; Median NS = 35.42px, S = 38.99px, *p* < 0.0001), major axis length (Figure 2E; Median NS = 12.19px, S = 12.93px, *p* < 0.0001), solidity (Figure 2F; Median NS = 0.7781px, S = 0.7833px, *p* = 0.0008), and mean radius (Figure 2G; Median NS = 1.254px, S = 1.331px, *p* < 0.0001) in S compared to NS animals. Additionally, there was a significant decrease in ipsilateral hemispheric section IBA1^+^ area eccentricity (Figure 2H; Median NS = 0.8382px, S = 0.8122px, *p* < 0.0001) in S animals compared to NS animals. Collectively, this analysis revealed an overall increase in IBA1^+^ area and transition to a larger (increase in area occupied and major axis length) and more amoeboid/rounded morphology (increases in solidity and decreases in eccentricity) in the ipsilateral hemisphere section of S animals compared to NS animals. Additional significant differences of IBA1^+^ areas between S and NS animals, as well as all other quantified stains, are detailed in the (Supplementary Table 1). PCA (Figures 2I–L) revealed a significant increase in ipsilateral section PC1-3 in S animals compared to NS animals (*p* < 0.0001).

**Figure 3:**
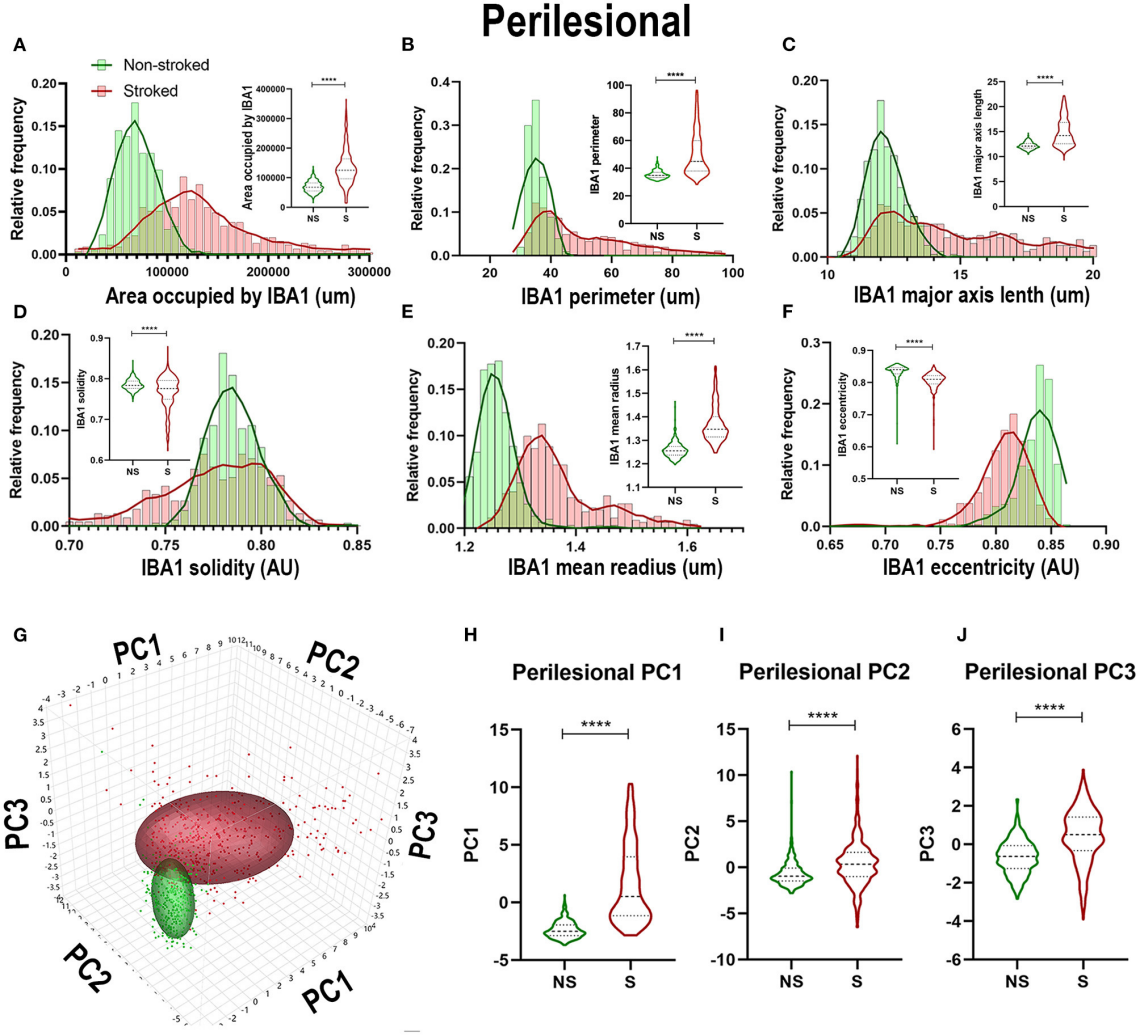
Stroke resulted in an amoeboid morphological transition of IBA1^+^ areas in perilesional tissue. Significant (*p* < 0.0001) increases in IBA1+ area occupied **(A)**, perimeter **(B)**, major axis length **(C)**, and mean radius **(E)** were observed in perilesional ROIs of S (*n* = 452 ROIs) animals compared NS (*n* = 304 ROIs) animals. There was a significant (*p* < 0.0001) decrease in solidity **(D)**, and eccentricity **(F)** of IBA1+ perilesional ROIs in S animals compared to NS animals. PCA **(G)** analysis showed a significant (*p* < 0.0001) increase in PC1-3 **(H–J)** in S compared to NS animals. ****p < 0.0001.

Quantification of morphological parameters of IBA1^+^ area in the ipsilateral perilesional ROIs (S; *n* = 453 ROIs and NS; *n* = 3 04 ROIs) showed similar findings to ipsilateral hemispheric section analysis. A significant increase in IBA1^+^ area occupied (Figure 3A; Median NS = 67,697px, S = 125,584px, *p* < 0.0001), IBA1^+^ area shape perimeter (Figure 3B; Median NS = 34.76px, S = 44.94px, *p* < 0.0001), major axis length (Figure 3C; Median NS = 12.12px, S = 14.22px, *p* < 0.0001), and mean radius (Median NS = 1.255px, S = 1.347px, *p* < 0.0001) as well as a significant decrease in solidity (Figure 3D; Mean NS = 0.7838, S = 0.7759, *p* < 0.0001) and eccentricity (Figure 3F; Mean NS = 0.8406, S = 0.8106 *p* < 0.0001) were shown in S compared to NS animals. IBA1^+^ area increased and transitioned to a more ameboid/rounded (decrease in eccentricity), larger (increase in major axis length, area occupied, and mean radius), morphology in the perilesional region of S animals compared to NS animals. There were additional significant differences between S and NS animals (Supplementary Table 2). PCA (Figures 3G–J) revealed a significant increase in perilesional PC1-3 between S and NS animals.

### GFAP^+^ Areas Transitioned to More Sprawled and Extended Morphology After Stroke

**Figure 4:**
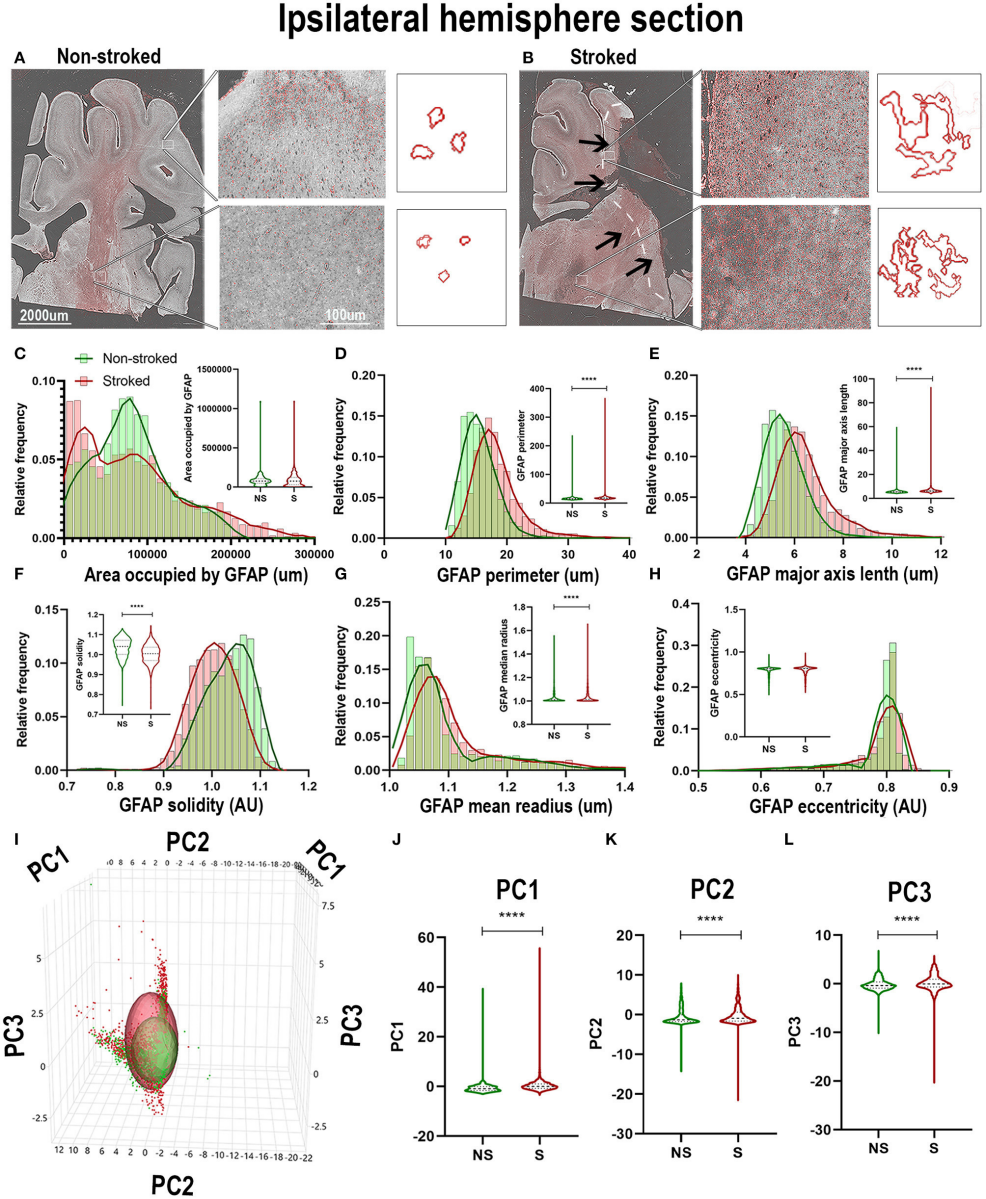
GFAP^+^ areas transitioned to more sprawled and extended morphology after stroke in ipsilateral tissue. Visual differences in density and location of GFAP^+^ areas were observed in ipsilateral sections, between S and NS animals **(A,B)**. Arrows indicate location of stroke lesion **(B)**. Significant (*p* < 0.0001) increases in perimeter **(D)**, major axis length **(E)**, solidity (**F**; *p* = 0.0003), and mean radius **(G)** were observed in hemispheric section ROIs of S (*n* = 1,848 ROIs) animals relative to NS (*n* = 1,668 ROIs) animals. There was a significant (*p* < 0.0001) decrease observed in GFAP^+^ area occupied **(C)** and solidity **(F)** of IBA1^+^ ROIs of in S animals compared to NS animals. No significant difference was seen in eccentricity **(H)**. PCA **(I)** analysis revealed a significant (*p* < 0.0001) difference in PC1-3 **(J–L)** in S animals compared to NS animals. ****p < 0.0001.

Positive area outlines revealed global visual differences between S and NS animals with regard to the density of GFAP^+^ areas in ipsilateral hemispheric sections (Figures 4A,B). Additionally, qualitative morphological evaluations revealed smaller, more rounded cells in NS animals and larger, more extended cells in S animals. These qualitative observations are supported by quantification of morphological parameters of GFAP^+^ in the ipsilateral hemisphere section, which showed significant differences between S (*n* = 1,848 ROIs) and NS (*n* = 1,668 ROIs) animals. A significant increase in GFAP^+^ area shape perimeter (Figure 4D; Median NS = 15.29px, S = 17.59px, *p* < 0.0001), major axis length (Figure 4E; Median NS = 5.492px, S = 6.236px, *p* < 0.0001), mean radius (Figure 4G; Median NS = 1.067px, S = 1.085px, *p* < 0.0001) and a significant decrease in solidity (Figure 4F; Median NS = 1.041px, S = 1.005px, *p* < 0.0001) were discovered in S compared to NS animals. Significant differences were not seen in ipsilateral section GFAP^+^ area occupied (Figure 4C; Median NS = 77,435px, S = px, p76,282, *p* = 0.5642) and eccentricity (Figure 4H; Median NS = 0.8009px, S = px, *p* < 0.8040, *p* < 0.0001) in S animals compared to NS animals. Collectively, GFAP^+^ area increased in S animals compared to NS animals (evidenced by an increase in perimeter, major axis length, and radius), and a transition to a less rounded and more ramified A1-like reactive astrocyte in S compared to NS animals (decrease in solidity) was observed. PC1-3 for GFAP^+^ areas significantly increased in ipsilateral hemispheric sections (*p* < 0.0001) in S animals compared to NS animals (Figures 4I–L).

**Figure 5:**
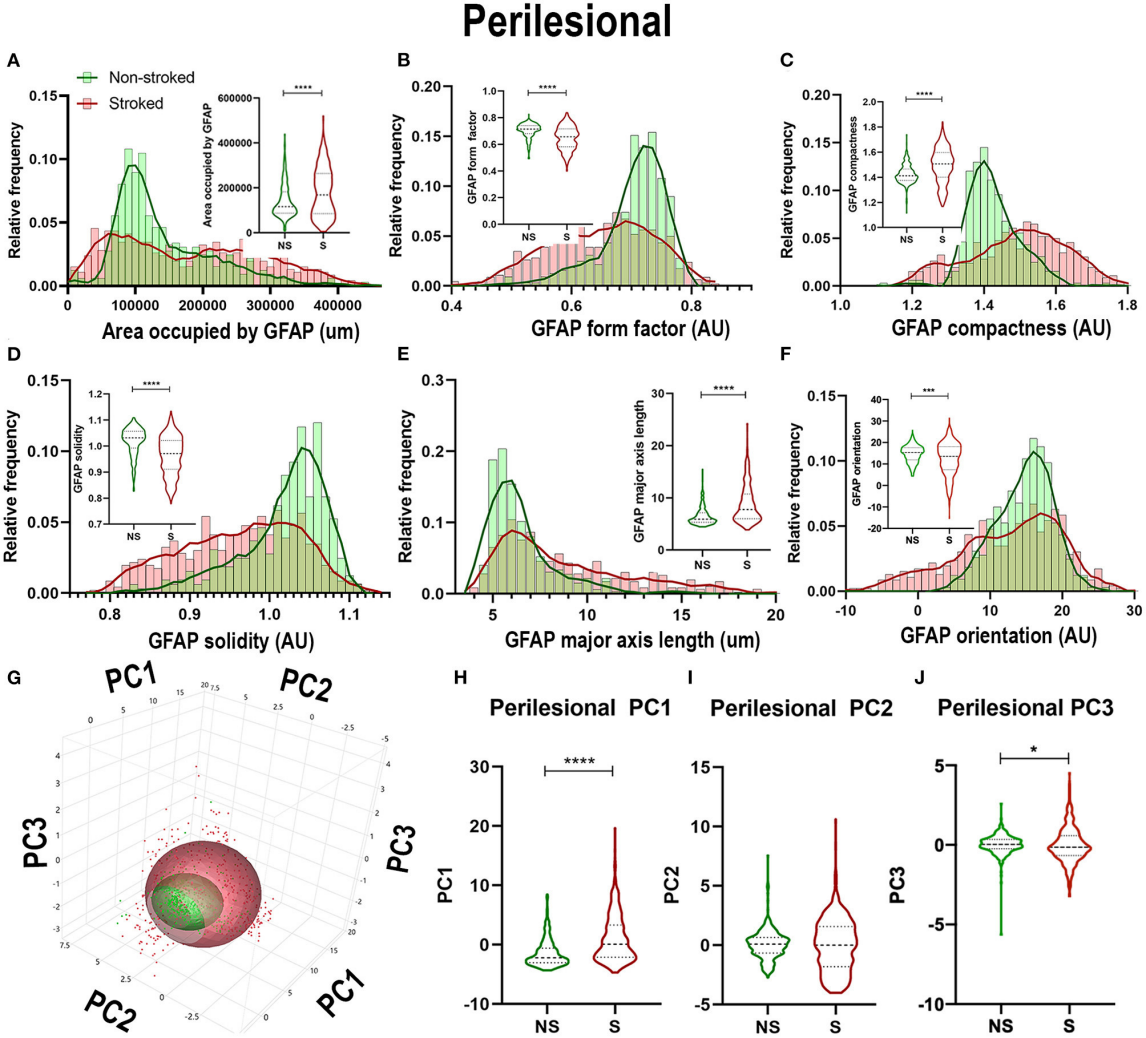
Stroke resulted in larger A1-like morphological changes of GFAP^+^ areas in perilesional tissue. Significant (*p* < 0.0001) increases in GFAP^+^ area occupied **(A)**, compactness **(C)**, and major axis length **(E)** were observed in ROIs of S (*n* = 461 ROIs) animals compared NS (*n* = 324 ROIs) animals. There was a significant decrease in form factor (**B**; *p* < 0.0001), solidity (**D**; *p* < 0.0001) and orientation (**F**; *p* = 0.0002) of IBA1^+^ ROIs in the perilesional region of S animals compared to NS animals. Upon PCA **(G)** analysis, a significant increase was observed in PC1 (**H**; *p* < 0.0001) and PC3 (**J**; *p* = 0.0162), but no significant difference was observed in PC2 **(I)**. *p < 0.05, ***p < 0.001, and ***p < 0.0001.

Quantification of morphological parameters of GFAP^+^ area in the perilesional region revealed a major difference from ipsilateral section ROI showing an increase in area occupied by GFAP^+^ area in S (*n* = 461 ROIs) relative to NS (*n* = 324 ROIs) animals (Figure 5A; Median NS = 116,517px, S = 168,980px, *p* < 0.0001). Similar to hemispheric section analysis, there was also a significant increase in compactness (Figure 5C; Median NS = 1.412px, S = 1.507x, *p* < 0.0001) and major axis length (Figure 5E; Median NS = 5.911px, S = 7.770px, *p* < 0.0001) and a significant decrease in form factor (Figure 5B; Median NS = 0.7143px, S = 0.6570px, *p* < 0.0001), solidity (Figure 5D; Median NS = 1.032px, S = 0.9710px, *p* < 0.0001), and orientation (Figure 5F; Median NS = 15.32px, S = 13.55px, *p* = 0.0002) suggesting a transition of GFAP^+^ areas to a more A1-like reactive astrocyte morphology in the perilesional region, as well as a greater area occupied by GFAP^+^ in S compared to NS animals. A significant increase in perilesional PC1 (*p* < 0.0001) and PC3 (*p* = 0.0162), but no significant differences in PC2 of GFAP^+^ ROIs was observed in perilesional tissue between S and NS animals (Figures 5G–J).

### NeuN^+^ Cells Transitioned to a Stressed Phenotype After Stroke

**Figure 6:**
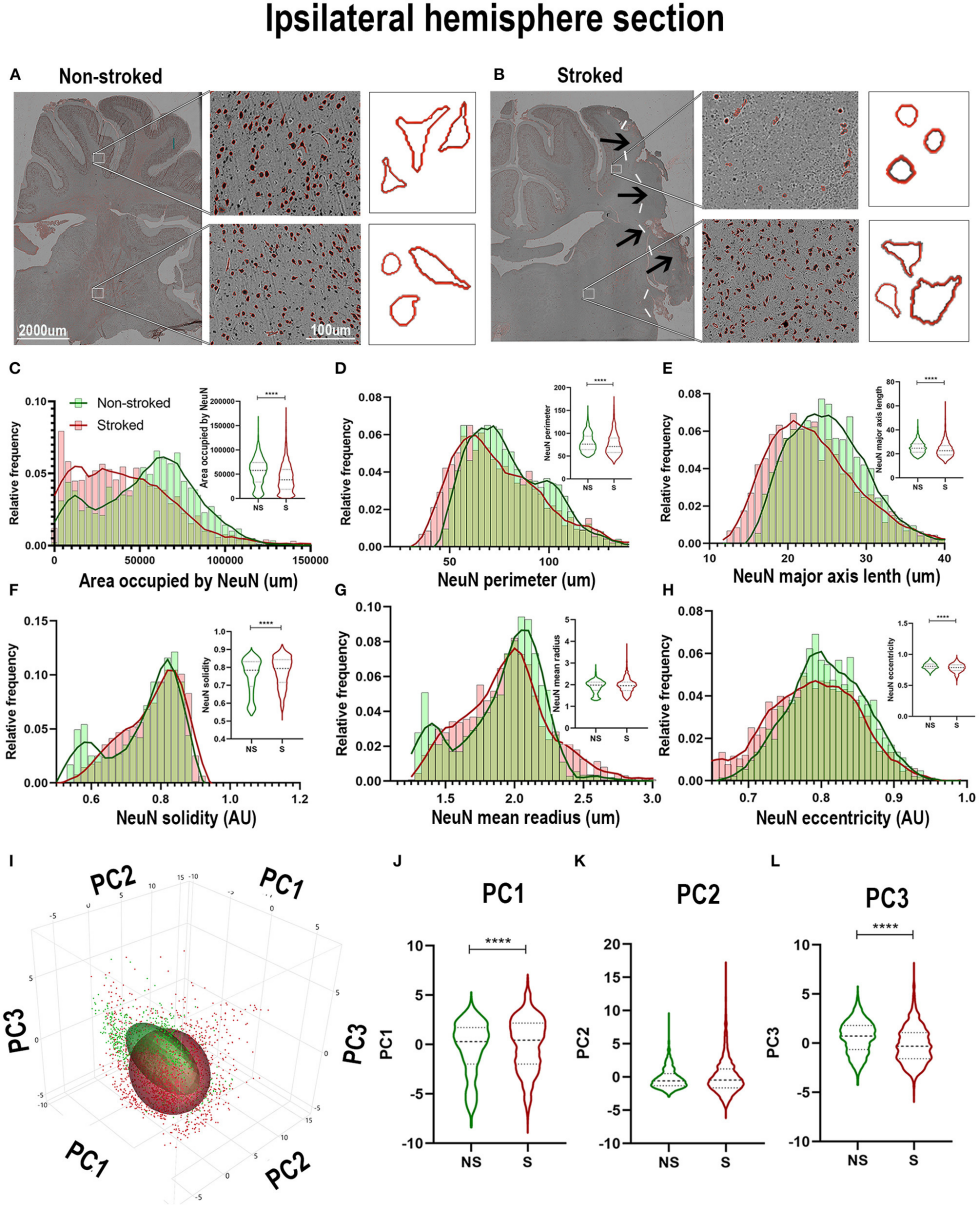
NeuN^+^ cells transitioned to a stressed phenotype after stroke in the ipsilateral hemisphere. Visual differences in density and location of NeuN^+^ areas in ipsilateral hemispheric sections were observed between NS and S animals **(A,B)**. Arrows indicate location of stroke lesion **(B)**. Significant (*p* < 0.0001) decreases in area occupied **(C)**, perimeter **(D)**, major axis length **(E)**, and eccentricity **(H)** were observed between ROIs of S (*n* = 2,230 ROIs) compared to NS (*n* = 1,476 ROIs) animals. There was a significant (*p* < 0.0001) increase observed in solidity **(F)** between NS and S animals. No significant difference was seen in mean radius **(G)**. PCA **(I)** analysis demonstrated a significant (*p* < 0.0001) increase in PC1 **(J)** and decrease in PC3 **(L)** of NeuN^+^ cells in S animals compared to NS animals, but no significant difference was observed in PC2 **(K)**. ****p < 0.0001.

While neuron loss is observed and expected at early time points following stroke, ongoing inflammation perpetuates a stressed/apoptotic state altering residual neuronal health. The nuclear morphology of these residual cells undergoing inflammatory stress even at chronic timepoints can indicate overall neuronal health, evidenced by a quantifiable transition to more apoptotic, stressed, morphology ($\frac{nuclear\;circumference}{form\;factor}$) (Eidet et al., 2014), or nuclear pyknosis and fragmentation (Zille et al., 2012) following stroke (Sairanen et al., 2005; Paltsyn et al., 2013). A qualitative loss of NeuN^+^ area was observed in perilesional regions in S compared to NS animals (Figures 6A,B). The ipsilateral section sections of S (*n* = 2,230 ROIs) were significantly decreased when compared to NS (*n* = 1,476 ROIs) in areas occupied by NeuN^+^ (Figure 6C; Median NS = 57,883px, S = 38,475px, *p* < 0.0001), NeuN^+^ area shape perimeter (Figure 6D; Median NS = 76.18px, S = 71.00px, *p* < 0.0001), major axis length (Figure 6E; Median NS = 24.78px, S = 22.65px, *p* < 0.0001), and eccentricity (Figure 6H; Median NS = 0.8065px, S = 0.7872px, *p* < 0.0001). A significant increase in solidity (Figure 6F; Median NS = 0.7841px, S = 0.7936px, *p* < 0.0001) was observed in S animals compared to NS animals. No significant difference was seen in mean radius (Figure 6G; Median NS = 1.980px, S = 1.955px, *p* = 0.3949). Collectively, there was an overall decrease in NeuN^+^ areas in S animals compared to NS animals and a transition to smaller, more compact and oblong cells (evideced by a decrease in perimeter, major axis length, compactness, and eccentricity) as is associated with nuclear morphological apoptosis. PCA (Figures 6I–L) revealed a significant (*p* < 0.0001) increase in ipsilateral section PC1 and a significant decrease in PC3 of NeuN^+^ areas in S compared to NS animals.

**Figure 7:**
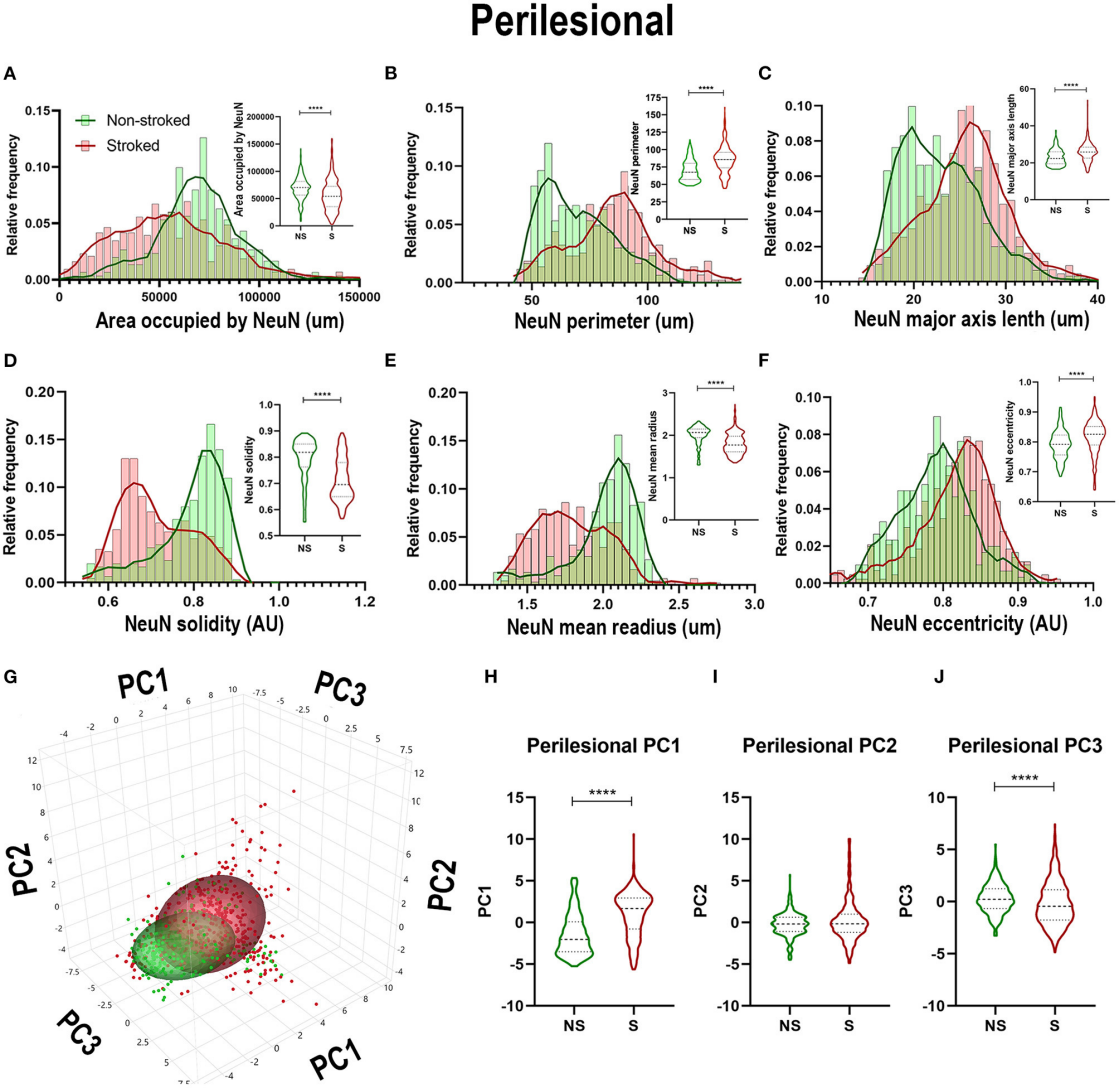
NeuN^+^ morphological changes in perilesional tissues differed from those in the larger ipsilateral hemisphere. Significant (*p* < 0.0001) decreases in NeuN^+^ area occupied **(A)**, perimeter **(B)**, solidity **(D)**, and mean radius **(E)** were observed in ROIs of S (*n* = 452 ROIs) animals compared NS (*n* = 354 ROIs) animals. Significant (*p* < 0.0001) increases in major axis length **(C)** and eccentricity **(F)** were observed in S animals compared to NS animals. PCA **(G)** analysis showed a significant (*p* < 0.0001) increase in PC1 **(H)** and decrease in PC3 (**J**; *p* < 0.0001) between S and NS animals, but no significant difference was observed in PC2 **(I)**. ****p < 0.0001.

Quantification of morphological parameters of NeuN^+^ cells in the perilesional region of S (*n* = 452 ROIs) and NS (*n* = 304 ROIs) animals demonstrated differences from ipsilateral section ROI quantification. While there was a significant decrease in area occupied by NeuN^+^ cells (Figure 7A; Median NS = 70,417px, S = 54,171px, *p* < 0.0001), as observed in hemisphere section analysis, there was an increase in NeuN^+^ perimeter (Figure 7B; Median NS = 67.52px, S = 85.73px, *p* < 0.0001), and eccentricity (Figure 7F; Median NS = 0.7921px, S = 0.8260px, *p* < 0.0001) contrary to directions seen in ipsilateral hemisphere analysis. There was also a decrease in NeuN^+^ solidity (Figure 7D; Median NS = 0.8184px, S = 0.6950px, *p* < 0.0001), and mean radius (Figure 7E; Median NS = 2.067px, S = 1.771px, *p* < 0.0001) which was opposite in direction to ipsilateral hemisphere analysis as well. PCA (Figures 7G–J) revealed a significant (*p* < 0.0001) increase in perilesional PC1 and a significant decrease in PC3 of S animals compared to NS animals.

### Disoriented FactorVIII^+^ Areas Increased in Coverage but Decreased in Size After Stroke

**Figure 8:**
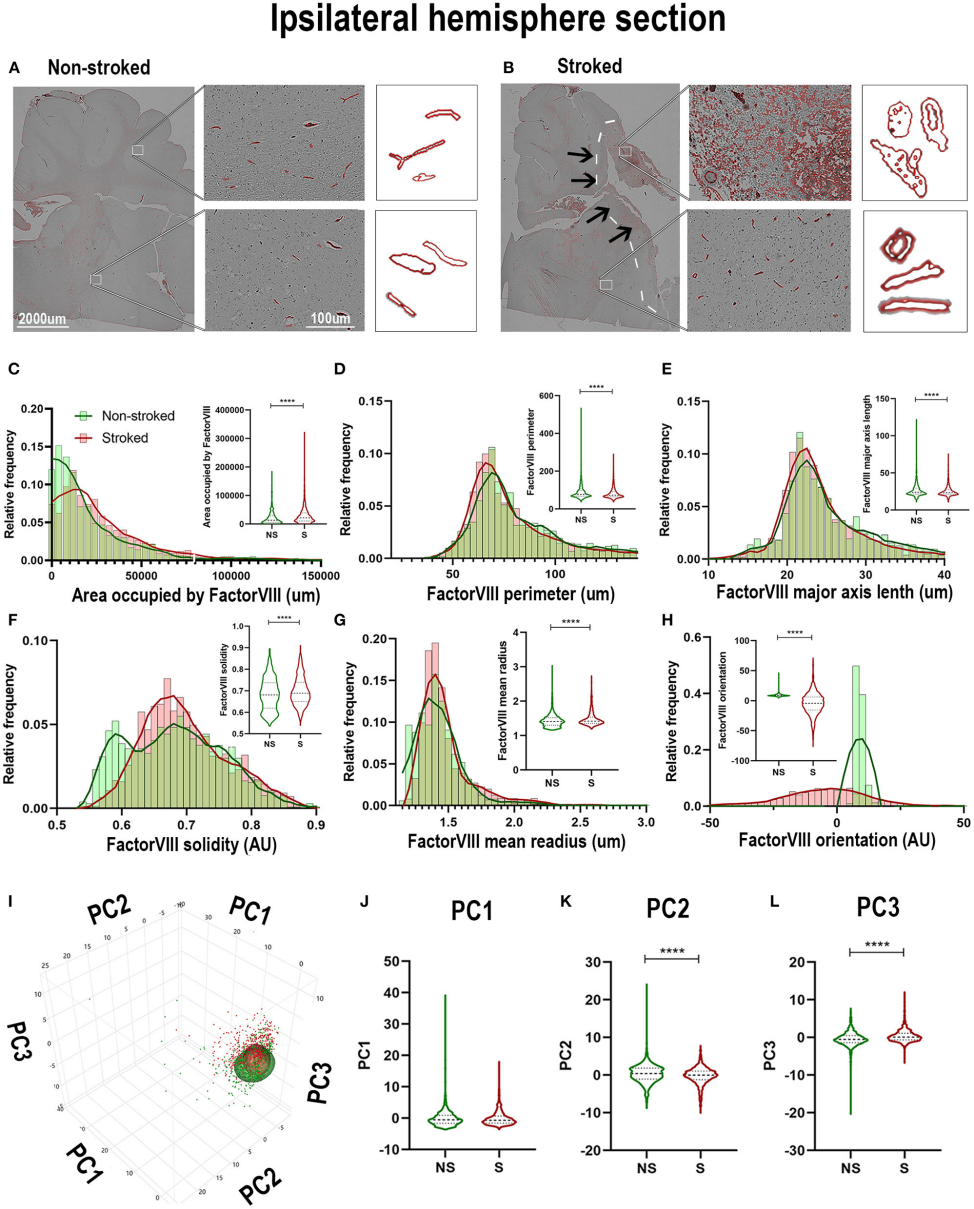
FactorVIII^+^ areas showed altered orientations and decreased size in the ipsilateral hemisphere following stroke. Visual differences in density and location of FactorVIII^+^ areas in ipsilateral hemispheric sections were observed between NS and S animals **(A,B)** (Barreto et al., 2011; Mittelbrunn et al., 2011). Arrows indicate location of stroke lesion **(B)**. Significant (*p* < 0.0001) increases in area occupied **(C)**, solidity **(F)**, and mean radius **(G)** were observed between ROIs of S (*n* = 1,228 ROIs) compared to NS (*n* = 1,584 ROIs) animals. There were also significant (*p* < 0.0001) decreases in perimeter **(D)**, major axis length **(E)**, and orientation **(H)** observed in between NS and S animals. PCA **(I)** analysis showed no difference in PC1 **(J)**, but a significant (*p* < 0.0001) decrease in PC2 **(K)** and increase in PC3 **(L)** of FactorVIII^+^ cells in S animals compared to NS animals. ****p < 0.0001.

FactorVIII has previously been utilized to identify vasculature in tissues and organs (Birner et al., 2001; Liang et al., 2007; Nag et al., 2019). In this study, qualitative evaluation of FactorVIII^+^ areas depict distribution and morphological differences in S animals compared to NS animals (Figures 8A,B). FactorVIII^+^ areas in perilesional regions in S animals displayed a dense distribution and unique morphological changes compared to NS animals. Quantification of morphological parameters of FactorVIII^+^ in the ipsilateral section sections revealed a significant increase in area occupied (Figure 8C; Median NS = 13,185px, S = 21,425px, *p* < 0.0001), solidity (Figure 8F; Median NS = 0.6806px, S = 0.6884px, *p* < 0.0001) and mean radius (Figure 8G; Median NS = 1.407px, S = 1.419px, *p* < 0.0001) between S (*n* = 1,584 ROIs) and NS (*n* =1, 128 ROIs) animals. Additionally, a significant decrease in area shape perimeter (Figure 8D; Median NS = 76.68px, S = 72.86px, *p* < 0.0001), major axis length (Figure 8E; Median NS = 23.98px, S = 23.30px, *p* < 0.0001), and orientation (Figure 8H; Median NS = 8.728px, S = −4.177px, *p* < 0.0001) were observed in S animal compared to NS animals. Collectively, this analysis showed an overall increase in smaller, more circular FactorVIII^+^ areas as well as a significant change in orientation in S animals. PCA (Figures 8I–L) revealed a significant (*p* < 0.0001) decrease in PC2 and a significant increase in PC3 of FactorVIII^+^ area in ipsilateral section ROIs of S animals compared to NS animals.

**Figure 9:**
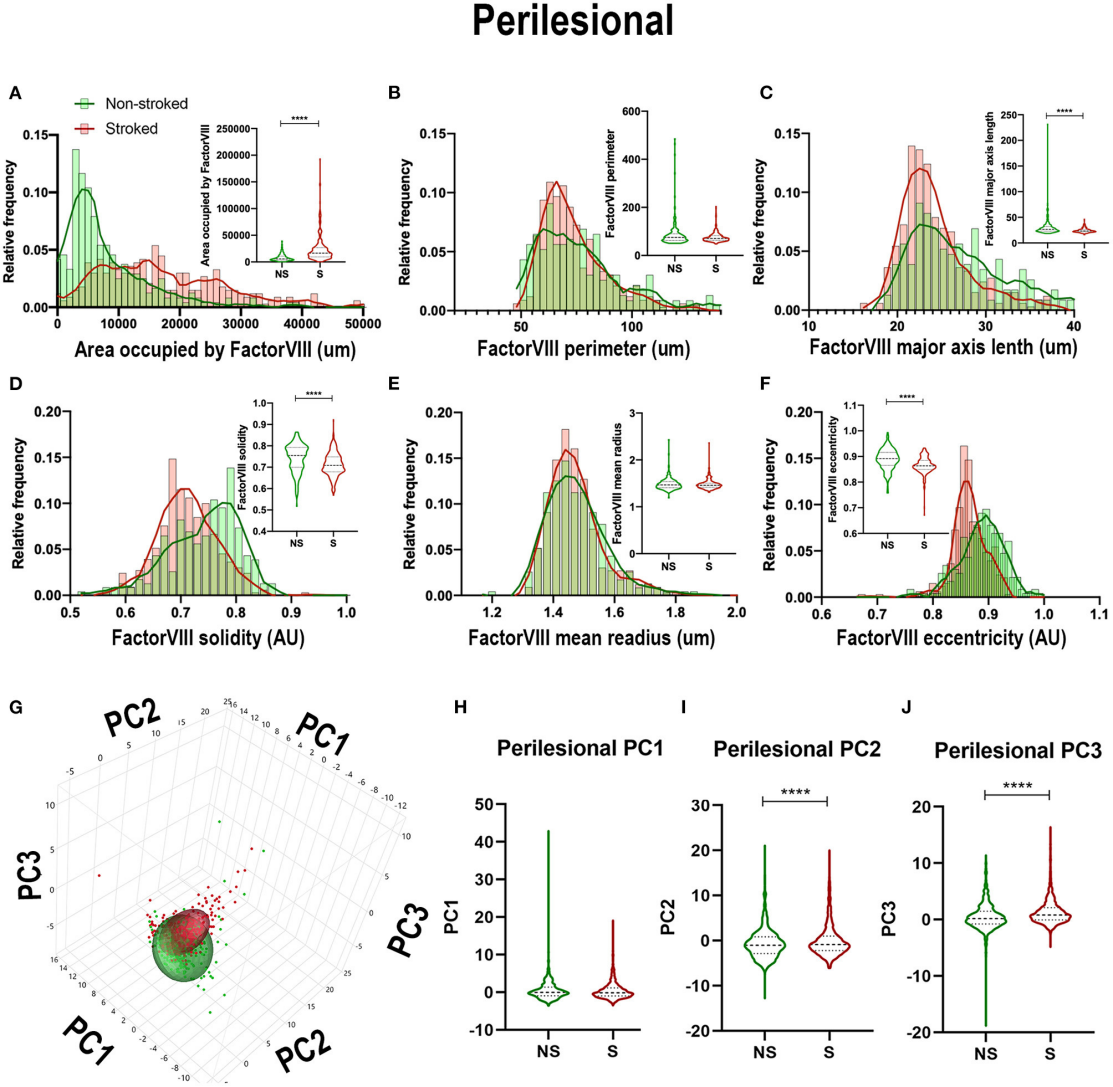
Stroke resulted in a decrease in size and solidity of FactorVIII^+^ areas in perilesional tissue. Significant (*p* < 0.0001) increase in NeuN^+^ area occupied **(A)**, and decreases in major axis length **(C)**, solidity **(D)**, and eccentricity **(F)** were observed in S (*n* = 334 ROIs) animals compared to NS (*n* = 240 ROIs) animals. No significant differences were seen in perimeter **(B)** and mean radius **(E)**. PCA **(G)** analysis showed no difference in PC1 **(H)**, but a significant (*p* < 0.0001) decrease in PC2 **(I)** and increase in PC3 **(J)** observed between S and NS animals. ****p < 0.0001.

Quantification of morphological parameters of FactorVIII^+^ area in the perilesional ROIs in NS (*n* = 240 ROIs) and S (*n* = 334 ROIs) animals showed similar findings to ipsilateral section ROI quantification. While in perilesional regions there was a significant increase in area occupied by FactorVIII^+^ (Figure 9A; Median NS = 5,667px, S = 16,598px, *p* < 0.0001) and a decrease in major axis length (Figure 9C; Median NS = 26.60 px, S = 23.38px, *p* < 0.0001) and eccentricity (Figure 9F; Median NS = 0.8917px, S = 0.8637px, *p* < 0.0001) in S animals compared to NS animals similar to ipsilateral section analysis, there was a contrasting decrease in solidity (Figure 9D; Median NS =0.7550px, S = 0.7086px, *p* < 0.0001). No significant differences were seen in area shape perimeter (Figure 9B; Median NS = 75.01px, S = 70.62px, *p* = 0.0869) and mean radius (Figure 9E; Median NS = 1.466px, S = 1.459px, *p* = 0.6348). PCA (Figures 9G–J) resulted in a significant (*p* < 0.0001) decrease in PC2 and increase in PC3 of S animals compared to NS animals in perilesional region analysis, similar to ipsilateral section analysis.

### Smaller Rounded DCX^+^ Areas Increased After pMCAO

There were no stark visual differences of DCX^+^ areas in the ipsilateral hemisphere section between S and NS animals (Supplementary Figures 1A,B). Quantification of morphological DCX^+^ parameters, however, did show a significant increase in area occupied (Supplementary Figure 1C; Median NS = 1,878px, S = 2,528px, *p* < 0.0001) and solidity (Supplementary Figure 1F; Median NS = 0.9328px, S = 0.9393px, *p* < 0.0001) of S (*n* = 2,004 ROIs) compared to NS (*n* = 1,241 ROIs). Additionally, a significant decrease in area shape perimeter (Supplementary Figure 1D; Median NS = 18.98px, S = 18.40px, *p* < 0.0001), and major axis length (Supplementary Figure 1E; Median NS = 6.994px, S = 6.921px, *p* = 0.0029), and mean radius (Supplementary Figure 1G; Median NS = 1.179px, S = 1.165px, *p* < 0.0002). There were no significant differences in orientation (Supplementary Figure 1H; Median NS = 0.1663px, S = 0.3648px, *p* = 0.6869). Collectively, ipsilateral section ROI analysis showed a significant increase in DCX^+^ area occupied with a transition to smaller (decrease in perimeter) rounder cells (increase in solidity) in S animals compared to NS animals. PCA (Supplementary Figures 1I–L) revealed a significant increase in PC1 (*p* < 0.0001) and PC2 (*p* < 0.0001) in DCX^+^ areas of ipsilateral section ROIs of S animals compared to NS animals.

Quantification of morphological parameters of DCX^+^ area in the ipsilateral perilesional ROIs of NS (*n* = 93 ROIs) and S (*n* = 128 ROIs) revealed differences from ipsilateral section ROI quantification. While in perilesional regions there was a significant increase in solidity (Supplementary Figure 2D; Median NS = 0.9348px, S = 0.9477px, *p* < 0.0001) and eccentricity (Supplementary Figure 2F; Median NS = 0.7673px, S = 0.7851px, *p* = 0.0008) and a decrease in perimeter (Supplementary Figure 2B; Median NS = 18.95px, S = 18.19px, *p* < 0.0001) of S compared to NS animals, as seen in ipsilateral section quantification, there was also a significant decrease in major axis length (Supplementary Figure 2C; Median NS = 6.875px, S = 6.528px, *p* < 0.0001), and mean radius (Supplementary Figure 2E; Median NS = 1.192px, S = 1.142px, *p* < 0.0001). There were no significant differences in area occupied by DCX^+^ (Supplementary Figure 2A; Mean NS = 3,162px S = 3,461px, *p* = 0.3344) of S compared to NS animals. PCA (Supplementary Figures 2G–J) revealed a significant decrease in PC1 (*p* < 0.0001) and PC2 (*p* = 0.0246) in S animals compared to NS animals in perilesional region analysis.

In addition to ipsilateral section and perilesional ROI quantification, DCX^+^ area in the subventricular zone (SVZ) was also quantified as the SVZ is a recognized neural stem cell niche in NS (Supplementary Figure 3A) and S (Supplementary Figure 3B) animals. DCX^+^ cells were also identified in the choroid plexus and included in the SVZ ROI quantification in NS (Supplementary Figure 3A) and S (Supplementary Figure 3B) animals. There was a significant difference of DCX^+^ morphology identified between S (*n* = 93 ROIs) and NS (*n* = 128 ROIs). There was a significant increase in area occupied by DCX^+^ (Supplementary Figure 3C; Median NS = 2,060px, S = 3,461px, *p* < 0.0001), major axis length (Supplementary Figure 3E; Median NS = 6.528x, S = 7.003px, *p* < 0.0001), solidity (Supplementary Figure 3F; Median NS = 0.9348px, S = 0.9477px, *p* < 0.0001), and eccentricity (Supplementary Figure 3H; Median NS = 0.7704px, S = 0.7836px, *p* = 0.0015). There was also a significant decrease in area shape perimeter (Supplementary Figure 3D; Median NS = 18.90px, S = 17.24px, *p* < 0.0001) and mean radius (Supplementary Figure 3G; Median NS = 1.192px, S = 1.142px, *p* < 0.0001). PCA (Supplementary Figures 3I–L) revealed a significant increase in PC2 (*p* = 0.0310) in S animals compared to NS animals in SVZ analysis.

### Relationships of Morphological Parameters Across Cell Types Altered in Stroked Yucatan Brain

**Figure 10:**
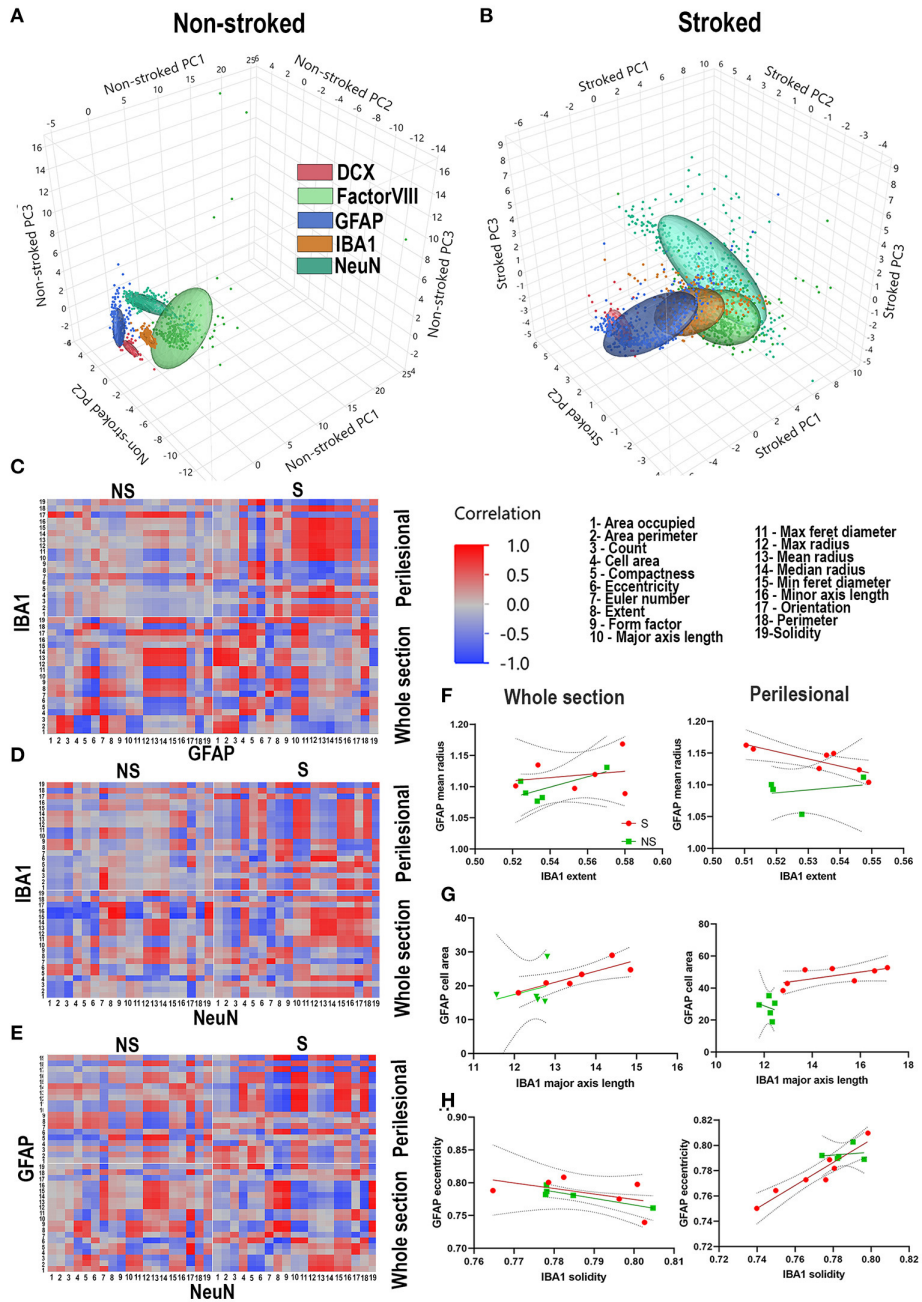
Stroke alters relationship of morphological parameters across cell types. PCA of NS and S ROIs labeled by DCX (red), FactorVIII (light green), GFAP (dark blue), IBA1 (tan), and NeuN (dark green). PCA ellipses for each stain are well-delineated in NS animals **(A)**, but not in S animals **(B)**. Heatmaps of each parameter measured 1-19 were generated for GFAP vs. IBA1 **(C)**, IBA1 vs. NeuN **(D)**, and NeuN vs. GFAP **(E)** for each treatment group (S and NS) as well as each ROI location (perilesional or hemispheric section). NS animals generally showed an increase in strength of correlations, positively or negatively, when comparing heatmaps of perilesional areas and hemispheric section areas. In S animals, there often was a complete reversal of correlation directionality between perilesional and hemispheric sections **(C–E)**. Linear correlations, representative of one square of the heatmap showed significant correlations of perilesional IBA1 extent and GFAP mean radius in S (*p* = 0.0220) but not NS animals (*p* = 0.6779) **(F)**; in hemispheric section IBA1 major axis length and GFAP cell area in S animals (*p* = 0.0289), but not NS animals (*p* = 0.6402); in hemispheric section ROIs of GFAP eccentricity and IBA1 solidity in NS animals (*p* = 0.0311), but not S animals (*p* = 0.3103) **(G)**; and in perilesional GFAP eccentricity and IBA1 solidity in S animals (*p* = 0.0011), but not NS animals (*p* = 0.7858) **(H)**.

PCA was conducted on NS and S ROIs labeled by DCX^+^ (red), FactorVIII^+^ (light green), GFAP^+^ (dark blue), IBA1^+^ (tan), and NeuN^+^ (dark green). While ellipses of NS animals for each stain are well-delineated (Figure 10A), PCA ellipses of S ROIs were not (Figure 10B).

The regional and inflammatory state-specific morphology of IBA1^+^, GFAP^+^, and NeuN^+^ cell types have been documented, making them excellent candidates to examine morphological correlations between these cell types using this novel HCI analysis approach. Heatmaps of each parameter measured (Gonzalez and Kolb, 2003; Wilhelmsson et al., 2006; Brown Craig et al., 2008; Thored et al., 2009; Chen et al., 2012; Karperien et al., 2013; Morrison and Filosa, 2013; Hovens et al., 2014; Avignone et al., 2015; Mestriner et al., 2015; Riordan et al., 2015; Savchenko et al., 2016; Sporns Peter et al., 2017; Heindl et al., 2018; Rayasam et al., 2018; Heo et al., 2020; Kaiser and West, 2020; Virani Salim et al., 2020) were generated for IBA1^+^ vs. GFAP^+^ (Figure 10C), IBA1^+^ vs. NeuN^+^ (Figure 10D), and NeuN^+^ vs. GFAP^+^ (Figure 10E) for hemispheric section and perilesional regions of S and NS groups. Multiple correlations of cell type to cell type S animal parameter comparisons (Figures 10C–E) were significantly divergent, often switching direction of correlation from NS animals regardless of location. In S animals, there were $\frac{26}{361}$ significant correlations of ipsilateral section and $\frac{49}{361}$ in perilesional tissue of IBA1^+^ morphology to GFAP^+^ morphology compared to only $\frac{6}{361}\;$and $\frac{9}{361}$ respectively in NS animals. In correlations of IBA1^+^ morphology to NeuN^+^ morphology in S animals, there were $\frac{21}{361}$ in ipsilateral section and $\frac{16}{361}$ in perilesional analysis compared to only $\frac{14}{361}$ and $\frac{2}{361\;}\;$in NS animals respectively. Lastly, in S animals there were $\frac{45}{361}\;$significant ipsilateral section and $\frac{64}{361}\;$perilesional correlations of GFAP^+^ morphology to NeuN^+^ morphology, compared to only $\frac{7}{361}\;$and $\frac{1}{361}$ in S animals. Ultimately in comparisons of all stains and locations, there were $\frac{221}{2166}$ significant correlations in S animals compared to $\frac{39}{2166}$ in NS animals.

Linear correlations, representative of one square of each heatmap, were evaluated to determine more specific relationships between morphological changes in stained areas (Figures 10F–H). While linear correlations between IBA1^+^ extent and GFAP^+^ mean radius are not significant between NS and S animals in ipsilateral section comparison, it is significant for S animals in perilesional comparisons (*p* = 0.0220) but not NS animals (*p* = 0.6779) (Figure 10F). In hemisphere section analysis, there was a significant linear correlation between IBA1^+^ major axis length and GFAP^+^ cell area in S animals (*p* = 0.0289), but not in NS animals (*p* = 0.6402). While there was no significant linear correlation between these parameters in either group in perilesional analysis, the x-intercepts of each group line were significantly different (*p* = 0.0119) (Figure 10G). There was a significant linear correlation in hemispheric section GFAP^+^ eccentricity and IBA1^+^ solidity in NS animals (*p* = 0.0311), but not S animals (*p* = 0.3103). In perilesional analysis, this was reversed and there was a significant linear correlation in GFAP^+^ eccentricity and IBA1^+^ solidity in S animals (*p* = 0.0011), but not NS animals (*p* = 0.7858) (Figure 10H). Quantified significant correlations between stains and treatment groups can be found in Supplementary Figure 10. These representative examples show some of the ways these correlations between cell types are regionally and injury dependent.

MRI analysis showed MCA territorial lesions with an average volume of 5.36 ± 3.68 cm^3^ in stroked animals (Supplementary Figures 9A,B). Simple linear regression revealed a significantly negative correlation between lesion volume and NeuN^+^ areas (Supplementary Figure 9E, *p* = 0.0126) and a significantly positive correlation between lesion volume and DCX^+^ areas (Supplementary Figure 9G, *p* = 0.083) in whole hemispheric analysis.

## Discussion

Standard histological analysis of cell morphology in most stroke studies have typically only examined a small subset of cellular features and relied on manual counting and analysis in a limited number of directly affected brain regions. Leveraging the advantages of HCI in a translational pig stroke model, semi-automated analysis of cell morphology was used to significantly increase the number of morphological features examined, eliminate the potential of user-bias, decrease processing time, and altogether increase histological analysis efficiency. Building from previous HCI studies, which detail computationally ascertained morphological changes in a single cell-type (Morrison and Filosa, 2013; Verdonk et al., 2016; Fernández-Arjona Md et al., 2017; Morrison et al., 2017; Heindl et al., 2018; York et al., 2018; Otxoa-de-Amezaga et al., 2019), this study revealed significant post-stroke morphological changes in 19 different cellular parameters in IBA1^+^, GFAP^+^, NeuN^+^, FactorVIII^+^, and DCX^+^ areas post-pMCAO. From all quantified parameters in both regions, perilesional and ipsilateral hemisphere section (19 parameters per region), a significant change in $\frac{38}{38}$ measured IBA1^+^ parameters, $\frac{34}{38}\;$GFAP^+^ parameters, $\frac{32}{38}$ NeuN^+^ parameters, $\frac{31}{38}$ FactorVIII^+^ parameters, and $\frac{28}{38}$ DCX^+^ parameters were observed in injured animals. While the p-values for many of these parameters indicated a strong significant difference between stroked and non-stroked animals, it is important to note that the large sample sizes utilized in this analysis may result in statistically significant results that may not be biologically significant. In light of this, analysis was not only directed at identifying individual parameter differences but also collective over-arching differences between respective cells in non-stroked and stroked animals through PCA. PCA demonstrated that these stroke-induced morphological changes were highly significant and suggested a stroke cell morphological fingerprint. Morphological analysis also revealed significant relationships between IBA1^+^, GFAP^+^, and NeuN^+^ areas with 1.8% of examined morphologies between these areas correlating pre-stroke and 10.2% correlating post-stroke. These correlative morphological changes suggest coordinated interactions associated with stroke pathological (e.g., immune and inflammatory responses) and recovery (e.g., neural reorganization) responses. However, additional studies modulating key cell signaling and molecular events are needed to further confirm the interplay between these cell types. The adapted HCI cellular morphological analysis approach showed that the stroke environmental effects led to comprehensive changes in cell morphology. This approach can be used as a platform technology to study unique cellular alterations due to injury or therapeutic intervention even in challenging systems such as the pig stroke model.

Identification of morphological correlations and relationships will enable the connection of cell specific interactions previously unknown. For example, here a positive correlation was observed between IBA1^+^ solidity (more round) and GFAP^+^ eccentricity (less round) in perilesional tissue in S animals, but not NS animals. While the morphological changes of activated astrocytes and microglia/macrophages have been described previously, in many studies these morphological changes are not directly quantified as in this study but rely on more qualitative observation (Price et al., 2006; Zhou et al., 2013; Huang et al., 2014; Hersh and Yang, 2018; Wang et al., 2019). The analysis utilized here not only quantifies activation-specific morphological changes through individual parameters, but also utilizes data reduction techniques to provide an overall value indicative of cellular change following stroke. This unbiased high throughput technique can be utilized to better quantify and demarcate discrete zones of activation stemming away from the focal area of ischemia. Furthermore, while the presence of DCX^+^ cells in the choroid plexus was unexpected in this study, it is not without precedence. Previously, neuroblasts co-labeled with DCX^+^ and BrdU^+^ were found to be present in the choroid plexus in chick embryos (Prasongchean et al., 2015). Additionally, Nestin and MAP2 expression has been shown to increase in rodent choroid plexus epithelial cells following exposure to traumatic human CSF, demonstrating the potential for neural blast cells to be located in this region following pro-inflammatory exposure (Hashemi et al., 2017). Moreover, this multi-cellular morphological analysis approach allowed for unique correlation analysis between post-stroke morphological changes in different cell types. While there was a small number of morphological correlations between cell types in the NS brain, there was a substantial increase in the number of significant correlations between cell types following stroke, indicating a strong relationship between the changing morphologies of immune and cytoarchitectural cells. In addition to understanding basic cell interactions in the stroke environment, the enhanced understanding of cell-to-cell morphological correlations may serve as biomarkers for key stroke molecular and cellular events or for therapeutic responses. Future studies utilizing this high content analysis platform are needed to more closely examine statistically significant changes in morphology for biological relevance.

As part of this work, we developed a generalizable morphological profiling pipeline that could serve as a platform for future histological stroke studies. When using manual processing approaches, stroke studies are often limited to a select range of features such as the number of positive cells (Ito et al., 2001; Hamzei Taj et al., 2016), cell body size (Zhan et al., 2008; Fumagalli et al., 2013), and number of extensions or processes (Masuda et al., 2011; Go et al., 2020). Previous semi-automated systems, such as Sholl concentric circle analysis used to identify cellular processes, ramification, and length of post-stroke GFAP^+^ astrocytes, did provide additional morphology-specific information, but lacked informative measures of ellipticity and other measures often associated with ischemia-induced immune cell alterations (Mestriner et al., 2015). These additional measures provide further insight into the continuous spectrum of cellular activation states outside of the previous bimodal classifications (Hamby and Sofroniew, 2010). Additionally, previous GFAP^+^ cell analyses also only provided information on 3 unique features, which is less relative to standard HCI studies (Carpenter et al., 2006; Caicedo et al., 2017). Traditional methods often rely on trained users to either manually count or trace cell outlines using image-based software, which can lead to bias in these measurements. In addition to an increase in bias, manual methods are more time consuming than more robust computational or semi-automated methods. In previous studies, automated processing of post-stroke microglia was shown to decrease an average manual counting time of 4–5 min per cell for a trained investigator to 5 min for the entire data-set of all cells (Heindl et al., 2018). Here, each individual pig (300 images per section, with 100s to 1,000s of cells per image) took ~1 h to analyze 1 stain spanning the ipsilateral hemisphere for 19 features. In contrast, the traditional manual quantification approach required up to 3.5 h per pig to analyze 1 stain in just the lesion border for a single feature (e.g., positive cells). Ultimately, when totaling the multiple stains per animal (5 stains) and multiple regions (2 regions) analyzed, semi-automation allowed for processing of approximately 160 million cells in 160 h for 16 animals, whereas analyzing the perilesional region alone for 5 stains would take ~280 h of manual counting. While this analysis was completed utilizing a standard desktop computer, the use of servers with faster processing speeds could drastically improve the efficiency of this semi-automated method. Current limitations of this study include the use of two-dimensional image analysis, whereas in the future, thicker sections can be utilized to capture more three-dimensional data. Given that the porcine brain is approximately 87x larger than a mouse brain, similar automated methods could increase the feasibility and efficiency of large animal studies or even be useful in histology-based clinical studies. As the large animal model is becoming increasingly valuable in the stroke field (Herrmann et al., 2019), the presented approach, which decreases bias by removing the element of human error and increasing regional sample size, is particularly valued for complex large animal experiments that have been historically prone to bias. The approaches shown herein can reduce bias which will improve translatability and allow the benefits of the large animal model to be used to its full potential. Further reduction of bias is vital to the success of large animal studies, including reports of lesion size variability, inclusion/exclusion criteria, randomization, and blinded assessment of outcome (Kringe et al., 2020).

Efficiencies afforded through this semi-automated analysis approach allowed for more robust quantification, interpretation, and elucidation of detailed biologically relevant cellular features. In this study, we evaluated 19 unique morphological features and determined significant morphological differences between IBA1^+^ areas in S and NS animals that demonstrated an increase in area occupied, perimeter, and major axis length that indicate a greater immune response in the perilesional area of S animals at 4 weeks post-pMCAO. While lesion volume may peak in early timepoints post-MCAO, rodent studies have demonstrated increases in inflammatory cells and cytokines in the post-acute stroke environment. For example, increases in neurotoxic factors and neuronal cell death persist from 7 to 25 days in young and aged rodent models of stroke (Aurel et al., 2007). In regard to chronic inflammation, rodent studies have shown that resident immune cells are persistently or sequentially activated at chronic time points with M1 microglia peaking at around 21 days post ischemic stroke (Jiang et al., 2020). Additionally, infiltrating cells such as CD4^+^ and CD8^+^ T-cells were found in the peri-infarct region of rodents 1 month after stroke in a transient occlusion model (Xie et al., 2019). While there is a growing body of evidence in rodent literature, there is a lack of data surrounding post-MCAO inflammation at later timepoints in the porcine literature. For these reasons, this later time point was chosen. For GFAP^+^ perilesional areas, we observed an opposite trend in the S animals compared to IBA1^+^, which aligns with an activated immune response morphology (e.g., sprawled and extended vs. rounded and ameboid, respectively) (Wang et al., 2019). This data also supports that analyzing multiple cell types in the post-stroke brain is informative because of unique interactions between cell types. For example, microglia are highly sensitive responders to central nervous system injuries with a relatively low activation threshold. Microglia release cytokines that influence the activation of GFAP^+^ cells resulting in glial scar formation and are critical for neuron-glial communication after ischemia (Price et al., 2006; Zhou et al., 2013; Huang et al., 2014; Yang et al., 2020). While these interactions between microglia, astrocytes (Hersh and Yang, 2018), neurons, and key factors in the stroke environment (e.g., cytokines) (Barreto et al., 2011; Becerra-Calixto and Cardona-Gómez, 2017; Li et al., 2020) have been characterized in the post-stroke brain, ischemia-induced morphological correlations between cells have not.

Ultimately, this study demonstrated for the first time dynamic morphological changes in microglia/macrophages, neurons, astrocytes, and vasculature after ischemic stroke and simultaneously found significant relationships between these morphological changes in these cell types in a preclinical gyrencephalic pig model. Understanding these morphological changes in specific cell populations in the stroke environment is critically important as it may provide insight into key cellular functions and biological responses. Future studies warrant further investigation into the dynamic morphological relationships between these cell types and specific signaling factors (e.g., inflammatory or neurodegenerative signaling) to better understand the interplay and organization of important injury and recovery processes and to ensure observed changes are not only statistically significant but biologically significant. Directly correlating more easily obtainable clinical data such as lesion volume from MRI to post-mortem histology findings offers significant contributions to further understanding the disease state at the cellular level (Annese, 2012). Here, we found a significant relationship between lesion volume and whole hemisphere section area occupied for both NeuN^+^ and DCX^+^ cells, indicating that an increase in DCX expression (neurogenesis) and a simultaneous decrease in NeuN expression (neuronal death) is correlated with overall stroke severity. Furthermore, correlating these histological findings to advanced MRI findings, such as functional MRI to observe neural reorganization, or to behavioral phenotyping would strengthen HCI data conclusions. The semi-automated HCI cellular morphology pipeline developed in this study is an unbiased tool that offers increased efficiency when evaluating cellular interactions in the stroke environment and offers a unique opportunity for therapeutic and biomarker discovery.

## Data Availability Statement

The raw data supporting the conclusions of this article will be made available by the authors, without undue reservation.

## Ethics Statement

The animal study was reviewed and approved by University of Georgia Institutional Animal Care and Use Committee (Protocol Number A2018 01-029-Y1-A5).

## Author Contributions

SES constructed CellProfiler pipelines and conducted histological imaging, processing, stitching, and histopathological analysis. SES and KS generated figures. KS, BJ, and EW conducted animal work. SES, KS, FW, and SLS wrote and edited the paper. SES, KS, EB, BJ, HK, SLS, and FW participated in experimental planning. JG performed OVX surgeries. HK and EB processed brains for histological submission. All authors contributed to the article and approved the submitted version.

## Conflict of Interest

SLS is a stockholder in Aruna Bio Inc and was a part-time employee of Aruna Bio Inc during the study. EB was a full-time employee of Aruna Bio Inc during the study. The remaining authors declare that the research was conducted in the absence of any commercial or financial relationships that could be construed as a potential conflict of interest.
