# Supplementary material for: Semi-Automated Cell and Tissue Analyses Reveal Regionally Specific Morphological Alterations of Immune and Neural Cells in a Porcine Middle Cerebral Artery Occlusion Model of Stroke
Source: Front Cell Neurosci. 2021 Jan 22;14:600441. doi: 10.3389/fncel.2020.600441 (PMC7862775; doi:10.3389/fncel.2020.600441)
Supplement: Supplementary file 1 [file Data_Sheet_1.PDF]

Supplementary Material

Supplementary Table 1. Definitions of all parameters measured. The 19 parameters measured for each cell are defined in the table.

Equations for parameter calculations are also included where applicable.

	Parameter	Definition	Equation
1	Area occupied	The number of positive pixels in a defined ROI	
2	Area shape area	The number of positive pixels in an object	
3	Compactness	The mean squared distance of the object's pixels from the centroid divided by the area	$\frac{\mu^2 \textit{ distance of objects pixels from centroid}}{\textit{area}}$ A perfect circle will have a compactness = 1 Irregular or objects with holes having values >1
4	Count	The number of positive objects in a specific ROI	
5	Eccentricity	The ratio of the distance between the foci of the ellipse and it's major axis length	$\frac{\textit{distance between the foci of the ellipse}}{\textit{major axis length}}$ A perfect circle = 0, whereas a line =1

6	Euler number	The number of objects in the region subtracted from the number of holes in those objects	
7	Extent	The proportion of positive pixels in the bounding box that are also in the region	$\frac{area}{volume\ of\ the\ bounding\ box}$
8	Form factor	Measure of circularity of object. Equal 1 for perfectly circular object	$\frac{4\pi \mathrm{Area}}{Perimeter^2}$
9	Major axis length	The length of the major axis of the positive ellipses that has the same normalized second central moments as the region	
10	Maximum radius	The maximum distance of any pixel in the object to the closest pixel outside of the object	
11	Maximum ferret diameter	The largest distance between 2 parallel lines tangent on either side of the object	
12	Mean radius	The mean distance of any pixel in the object to the closest pixel outside of the object	
13	Median radius	The median distance of any pixel in the object to the closest pixel outside of the object	

14	Min ferret diameter	The smallest distance between 2 parallel lines tangent on either side of the object	
15	Minor axis length	The length in pixels of the minor axis of the ellipse that has the same normalized second central movements as the region	
16	Orientation	The angle between the x-axis and the major axis of the ellipse that has the same second-moments as the region between -90 and +90	
17	Perimeter	The additive number of pixels around the boundary of positive areas total for each ROI	
18	Perimeter shape area	The number of pixels around the boundary of each positive area	
19	Solidity	The proportion of positive pixels in the convex hull which are also in the object	$\frac{ObjectArea}{ConvexHullArea}$

Supplementary Table 2. Morphological parameters for each stain by location. All parameter means for NS and S groups, and p-values not included in figures are included in the table here. P-values < 0.0001 are bold and italicized and values <0.05 are bolded.

Parameter	Location	IBA1 NS median	IBA1 S median	IBA1 p-value	GFAP NS median	GFAP S median	GFAP p-value	NeuN NS median	NeuN S median	NeuN p-value	FactorVIII NS median	FactorVIII S median	FactorVIII p-value	DCX NS median	DCX S mean	DCX p-value
Count	Hemisphere section	1704	1826	0.0017	6891	4996	0.1638	381.0	262.0	<0.001	100.0	171.5	<0.0001	86.00	114.0	<0.0001
	Perilesional	1597	2010	<0.0001	6370	5031	<0.0001	466.0	343.0	<0.0001	46.00	145.5	<0.0001	135.00	172.0	0.0044
Compactness	Hemisphere section	1.848	1.749	<0.0001	1.376	1.443	<0.0001	1.964	1.818	<0.0001	2.920	2.654	<0.0001	1.350	1.351	0.7441
	Perilesional	1.833	1.747	<0.0001	1.412	1.507	<0.0001	1.700	2.164	<0.0001	3.061	2.564	<0.0001	1.301	1.344	0.0218
Perimeter of area occupied (pixels)	Hemisphere section	49,421	60,676	<0.0001	57,863	54,608	<0.0001	24,219	15,708	<0.0001	7,383	11,332	<0.0001	1,269	1,596	<0.0001
	Perilesional	45,904	76,312	<0.0001	75,272	103,089	<0.0001	26,861	23,605	<0.0001	3,200	9,213	<0.0001	2,123	2,311	0.0399
Area of positive shape areas (pixels)	Hemisphere section	42.91	52.81	<0.0001	13.68	17.27	0.0011	157.5	151.1	<0.0001	111.1	111.0	0.0741	20.731	19.73	0.0025
	Perilesional	42.39	64.85	<0.0001	18.42	27.22	<0.0001	151.72	151.1	0.1678	117.6	111.8	0.2436	21.19	17.661	<0.0001
Euler number	Hemisphere section	0.9571	0.9228	<0.0001	0.9845	0.9762	0.5282	1.000	1.000	0.7072	0.6042	0.6579	0.0026	0.9873	0.9930	<0.0001
	Perilesional	0.9638	0.8427	<0.0001	0.9503	0.9181	<0.0001	1.000	1.000	0.71887	0.7388	0.7222	0.2290	0.9885	0.9950	<0.0001
Extent	Hemisphere section	0.51719	0.5333	<0.0001	0.6661	0.6509	<0.0001	0.5355	0.55511	<0.0001	0.4112	0.4291	<0.0001	0.6616	0.6656	0.0124
	Perilesional	0.5265	5313	0.0181	0.6460	0.6259	<0.0001	0.5640	0.4572	<0.0001	0.4223	0.4519	<0.0001	0.6783	0.6767	0.4927
Form Factor	Hemisphere section	0.4734	0.4893	<0.0001	0.7454	0.7107	<0.0001	0.4820	0.4779	0.0005	0.2837	0.3060	<0.0001	0.6943	0.7006	0.0007
	Perilesional	0.4821	0.4793	0.0034	0.7413	0.6570	<0.0001	0.5582	0.3342	<0.0001	0.4282	0.4449	<0.0001	0.7522	0.7155	0.7522
Max ferret diameter	Hemisphere section	10.90	11.72	<0.0001	4.395	5.149	<0.0001	23.91	21.814	<0.0001	22.00	21.33	0.0002	6.068	5.951	0.011

(pixels)	Perilesional	10.77	13.09	***<0.0001***	4.814	6.714	***<0.0001***	21.82	24.53	***<0.0001***	24.333	21.41	***<0.0001***	5.975	5.541	***<0.0001***
Maximum Radius	Hemisphere section	2.251	2.510	***<0.0001***	1.364	1.4589	***<0.0001***	4.571	4.469	0.8092	2.853	2.893	***<0.0001***	1.861	1.810	***0.0002***
(pixels)	Perilesional	2.249	2.568	***<0.0001***	1.411	1.600	***<0.0001***	4.782	4.085	***<0.0001***	2.952	2.983	0.2068	1.890	1.724	***<0.0001***
Median radius	Hemisphere section	1.033	1.081	***<0.0001***	1.010	1.013	***<0.0001***	1.758	1.735	0.5548	1.159	1.228	***<0.0001***	1.040	1.033	0.2247
(pixels)	Perilesional	1.033	1.092	***<0.0001***	1.015	1.020	***=0.0001***	1.843	1.518	***<0.0001***	1.215	1.201	**0.0311**	1.049	1.024	**0.0005**
Min ferret diameter	Hemisphere section	5.259	5.922	***<0.0001***	1.987	2.361	***<0.0001***	10.93	10.64	**<0.0001**	8.586	8.736	0.2778	2.992	2.869	***0.0002***
(pixels)	Perilesional	5.198	6.655	***<0.0001***	2.262	3.151	***<0.0001***	10.81	11.25	***<0.0001***	7.607	8.613	***<0.0001***	3.059	2.664	***<0.0001***
Minor axis length	Hemisphere section	5.703	6.382	***<0.0001***	2.612	2.974	***<0.0001***	11.01	11.74	***<0.0001***	8.728	8.853	0.3636	3.756	3.618	***<0.0001***
(pixels)	Perilesional	5.623	7.020	***<0.0001***	2.869	3.680	***<0.0001***	10.81	11.25	***<0.0001***	7.750	8.812	***<0.0001***	3.809	3.431	***<0.0001***
Orientation	Hemisphere section	-2.274	-1.1443	***0.0006***	16.29	13.45	***<0.0001***	-6.822	-2.410	***<0.0001***	8.728	-4.177	***<0.0001***	0.1663	0.3648	0.6869
	Perilesional	-2.216	-0.3821	***<0.0001***	15.32	13.55	***0.0002***	-9.170	1.289	***<0.0001***	-5.192	3.260	***<0.0001***	0.5714	3.543	***0.0015***
Total significant	Hemisphere section			*12 out of 12*			*10 out of 12*			*9 out of 12*			*9 out of 12*			*10 out of 12*
	Perilesional			*12 out of 12*			*12 out of 12*			*10 out of 12*			*9 out of 12*			*10 out of 12*

Ipsilateral hemisphere section

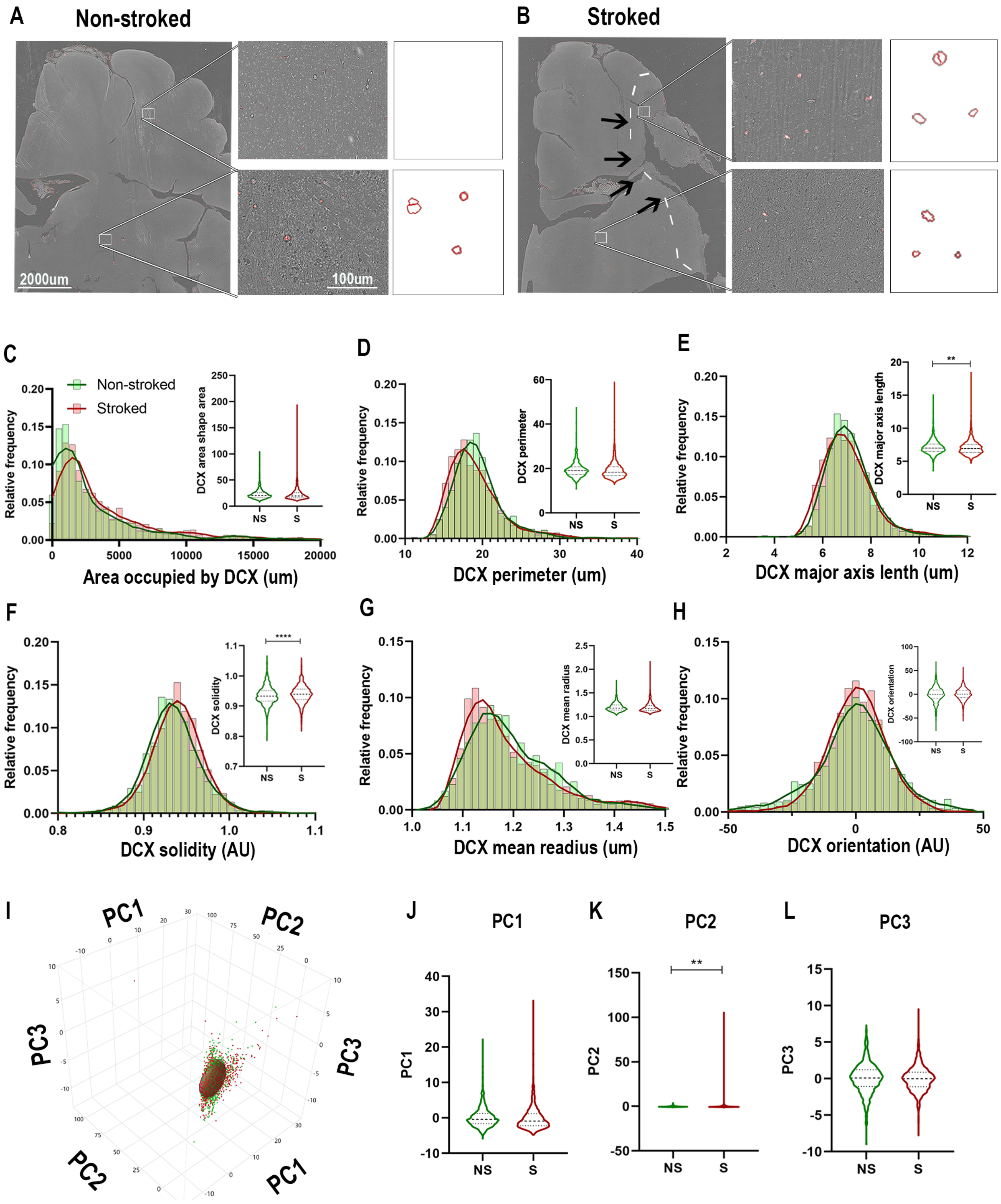

Supplementary Figure 1. Stroke resulted in an increase in DCX$^+$ areas with smaller perimeter in the ipsilateral hemisphere. Visual differences in density and location of DCX$^+$ areas in ipsilateral hemispheric sections were observed between NS and S animals (**A-B**). Arrows indicate location of stroke lesion (**B**). Significant ($p<0.0001$) increases in area occupied (**C**) and solidity (**F**) were observed between S (n=1,241 ROIs) and NS (n=2,004 ROIs) animals. There was a significant ($p<0.0001$) decrease in perimeter (**D**), major axis length (p=0.0029), and (**E**), mean radius (p=0.3061) (**G**) observed between S and NS animals. There was no significant difference in orientation (p=0.3016) (**H**). PCA **(I)** analysis revealed a significant increase in PC1 (**J**) and PC2 **(K;** p=0.0025) in DCX$^+$ cells in S animals compared to NS animals.

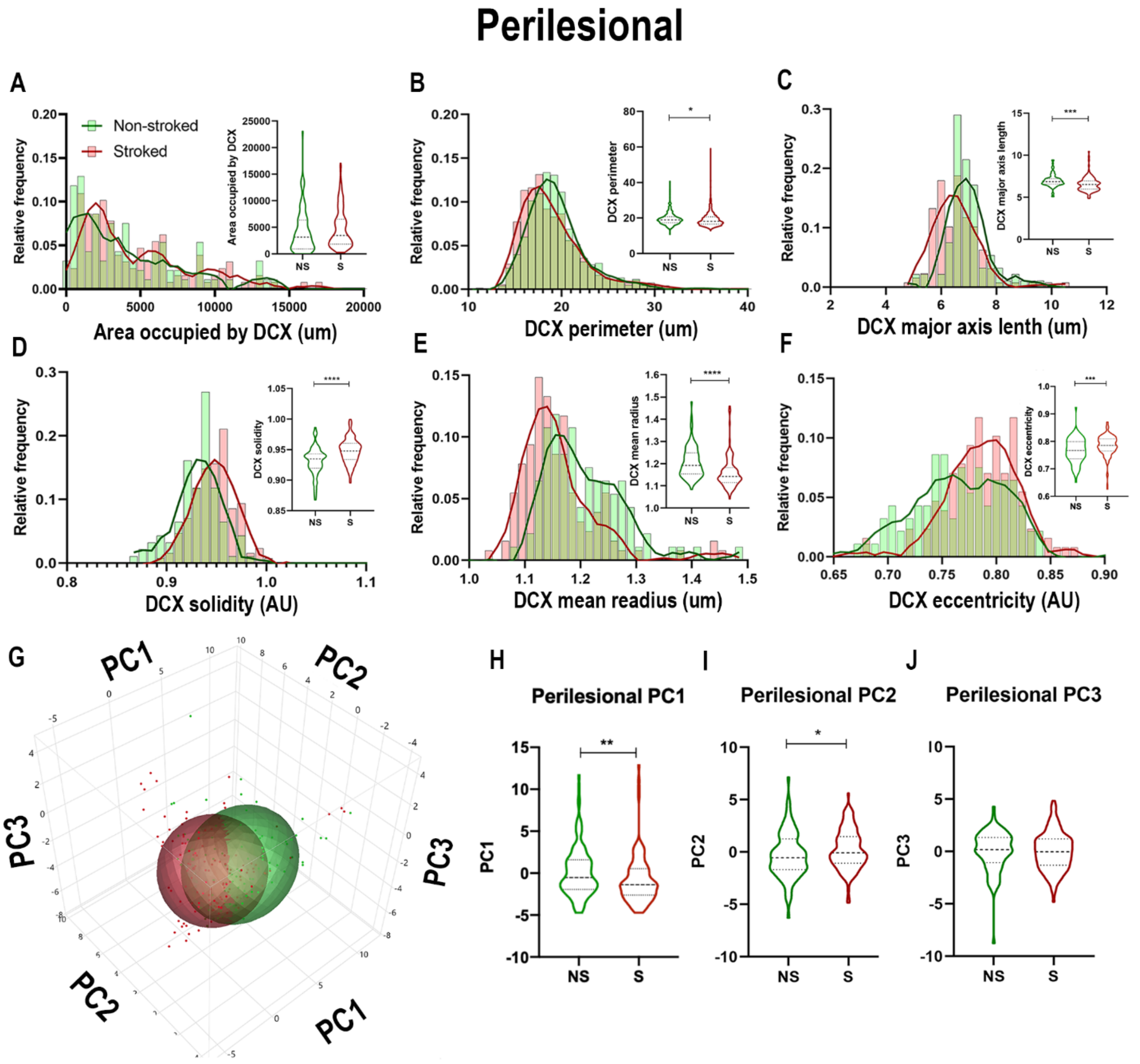

Supplementary Figure 2. Stroke resulted in decreased size of DCX$^+$ areas in perilesional tissue. Significant ($p<0.0001$) decreases in perimeter (**B**), major axis length (**C**), and mean radius (**E**) and significant increases in solidity (**D**; $p<0.0001$) and eccentricity (**F**; $p=0.0008$) were observed in S (n=128 ROIs) animals compared NS (n=93 ROIs) animals. There was no significant difference in DCX$^+$ area occupied (**A**). PCA (**G**) analysis showed a significant

decrease in PC1 (**H**; $p<0.0001$), increase in PC2 (**I**; $p=0.0246$), and no difference in PC3 (**J**) between S and NS animals.

frontiers

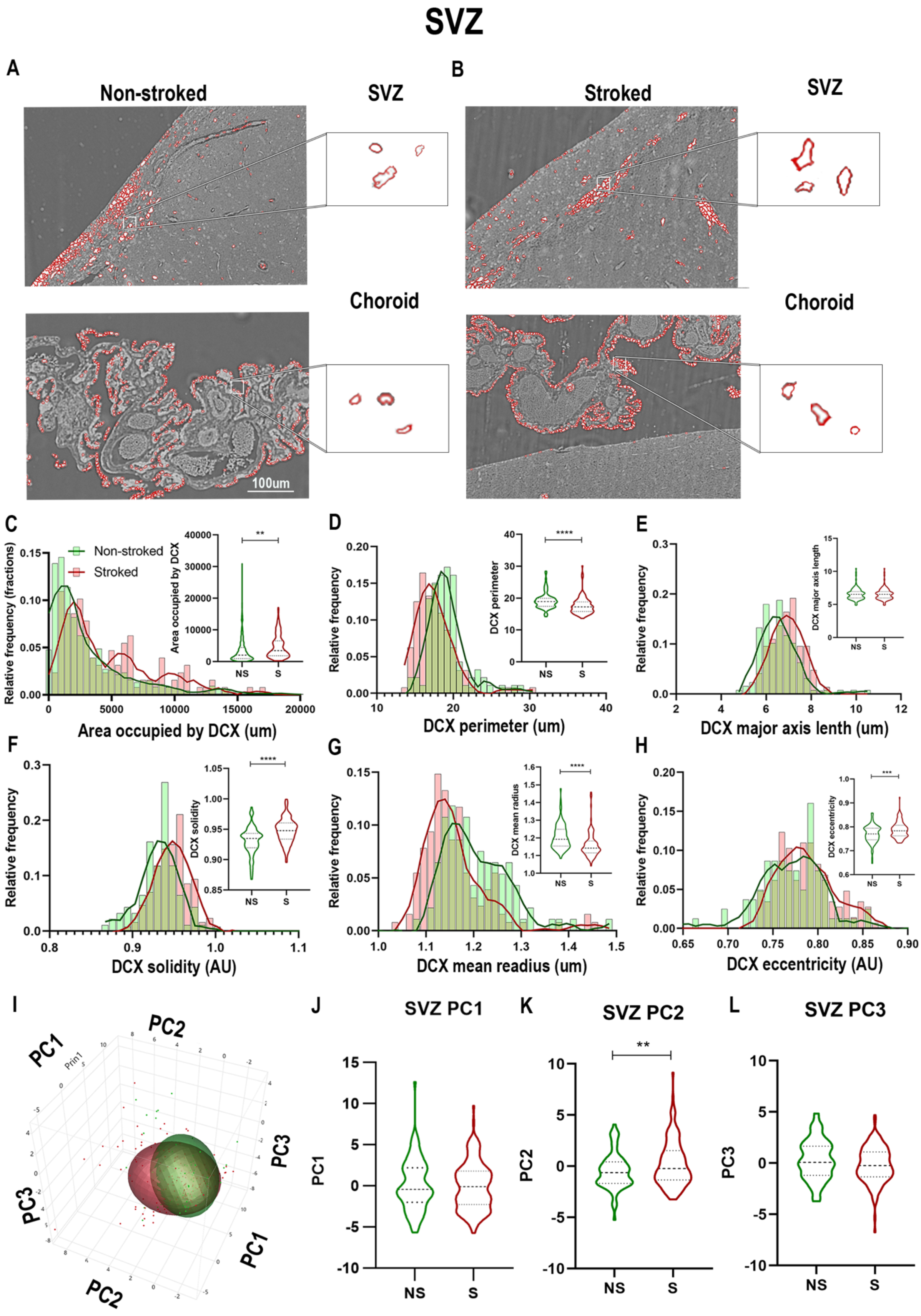
SVZ
A
Non-stroked
SVZ
Choroid
100um
B
Stroked
SVZ
Choroid
C
Non-stroked
Stroked
Relative frequency (fractions)
Area occupied by DCX
Area occupied by DCX (um)
NS
S
**
D
Relative frequency
DCX perimeter
DCX perimeter (um)
****
E
Relative frequency
DCX major axis length
DCX major axis lenth (um)
F
Relative frequency
DCX solidity
DCX solidity (AU)
****
G
Relative frequency
DCX mean radius
DCX mean readius (um)
****
H
Relative frequency
DCX eccentricity
DCX eccentricity (AU)
***
I
PC1
PC2
PC3
J
SVZ PC1
PC1
K
SVZ PC2
PC2
**
L
SVZ PC3
PC3
NS
S

Supplementary Figure 3. Stroke results in an increase of DCX$^+$ areas in the SVZ. Visual differences in morphology of DCX$^+$ areas in the SVZ and the choroid plexus were observed between NS (n=128 ROIs) and S (n=93 ROIs) animals (**A-B**). Significant ($p<0.0001$) increases in DCX$^+$ area occupied (**C**), major axis length (**E**), solidity (**F**), and eccentricity (**H**) were observed in S animals compared NS. There was also a significant ($p<0.0001$) decrease in perimeter (**D**) and mean radius (**G**) in S animals compared to NS animals. PCA (**I**) analysis showed no difference in PC1 (**J**) or PC3 (**L**), but a significant ($p<0.0001$) increase in PC2 (**K**) between S and NS animals.

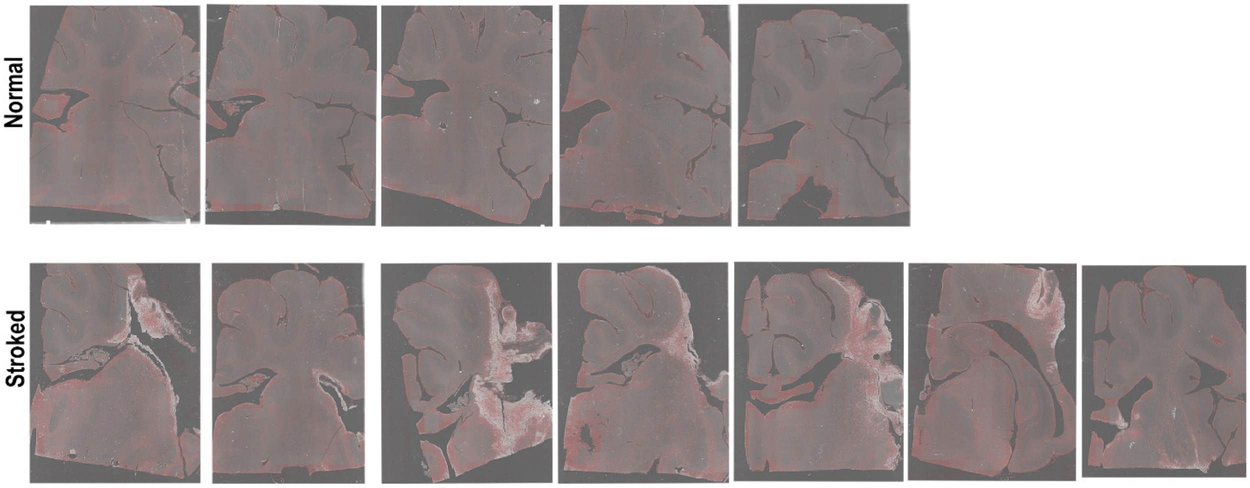

Supplementary Figure 4. Processed and stitched IBA1^{+} sections of normal and stroked animals. IBA1^{+} areas in each ROI (red) reveal concentrations of microglia/macrophages in ipsilateral sections of normal animals (top row), and after pMCAO stroke (bottom row).

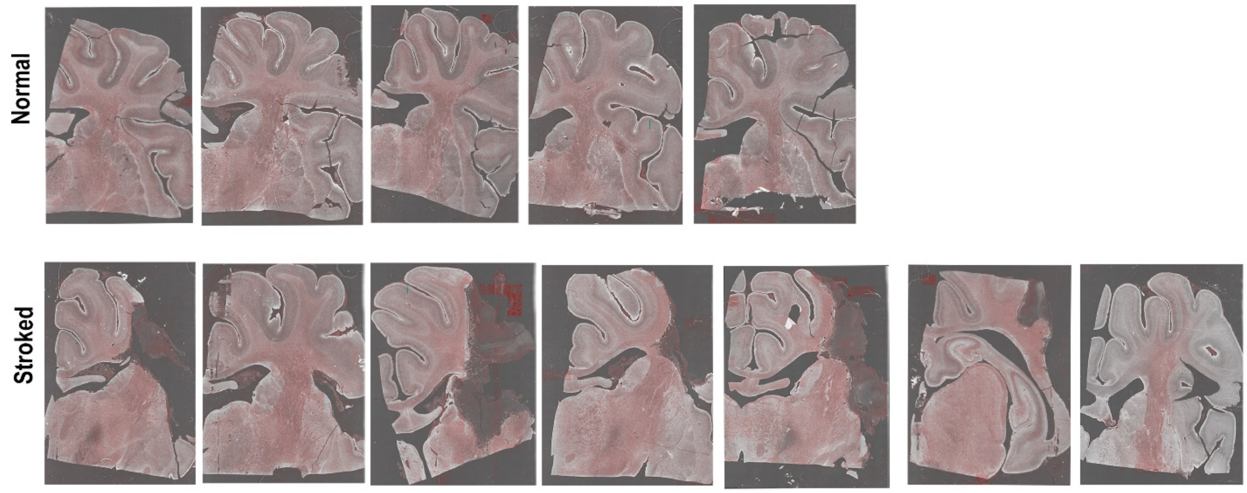

Supplementary Figure 5. Processed and stitched GFAP$^+$ sections of normal and stroked animals. GFAP$^+$ areas in each ROI (red) reveal concentrations of astrocytes in ipsilateral sections of normal animals (top row), and after pMCAO stroke (bottom row).

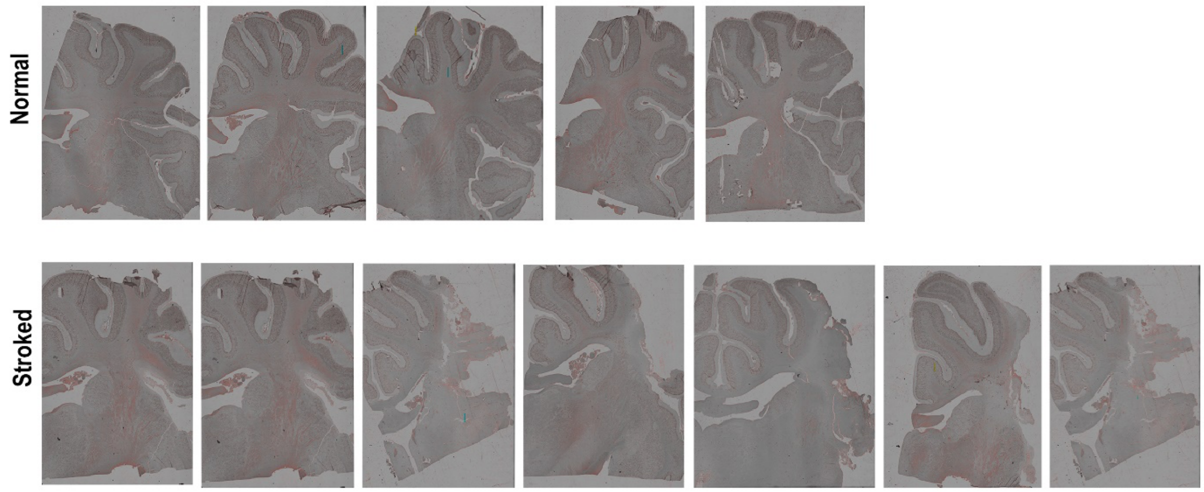

Supplementary Figure 6. Processed and stitched NeuN$^+$ sections of normal and stroked animals. NeuN$^+$ areas in each ROI (red) reveal concentrations of neuronal nuclei in ipsilateral sections of normal animals (top row), and after pMCAO stroke (bottom row).

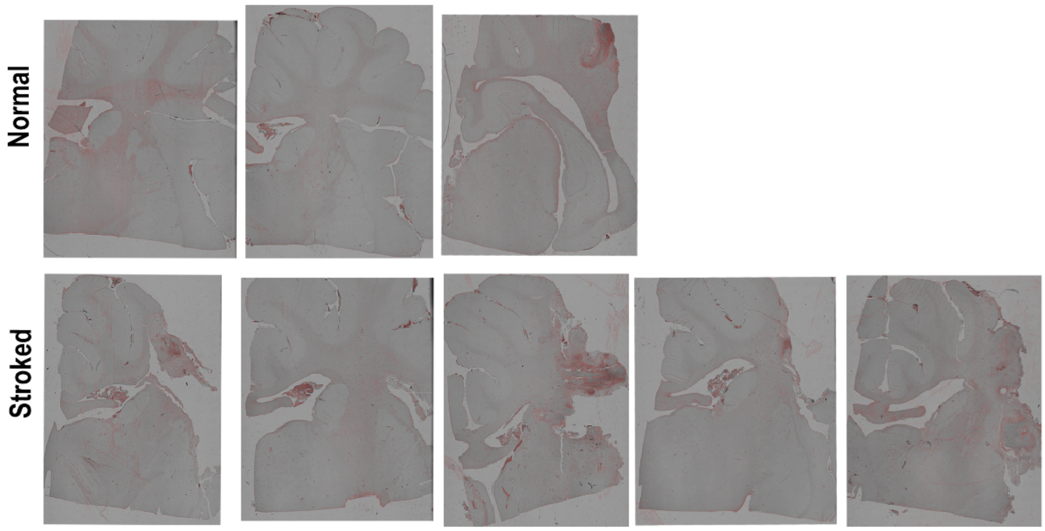

Supplementary Figure 7. Processed and stitched FactorVIII$^+$ sections of normal and stroked animals. FactorVIII$^+$ areas in each ROI (red) reveal concentrations of vasculature in ipsilateral sections of normal animals (top row), and after pMCAO stroke (bottom row).

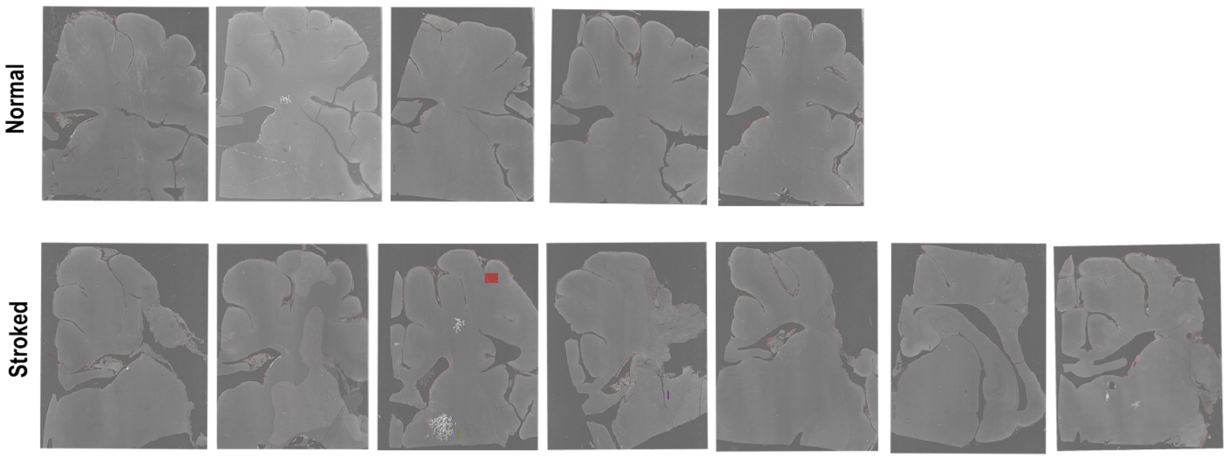

Supplementary Figure 8. Processed and stitched DCX$^+$ sections of normal and stroked animals. DCX$^+$ areas in each ROI (red) reveal concentrations of neural progenitors in ipsilateral sections of normal animals (top row), and after pMCAO stroke (bottom row).

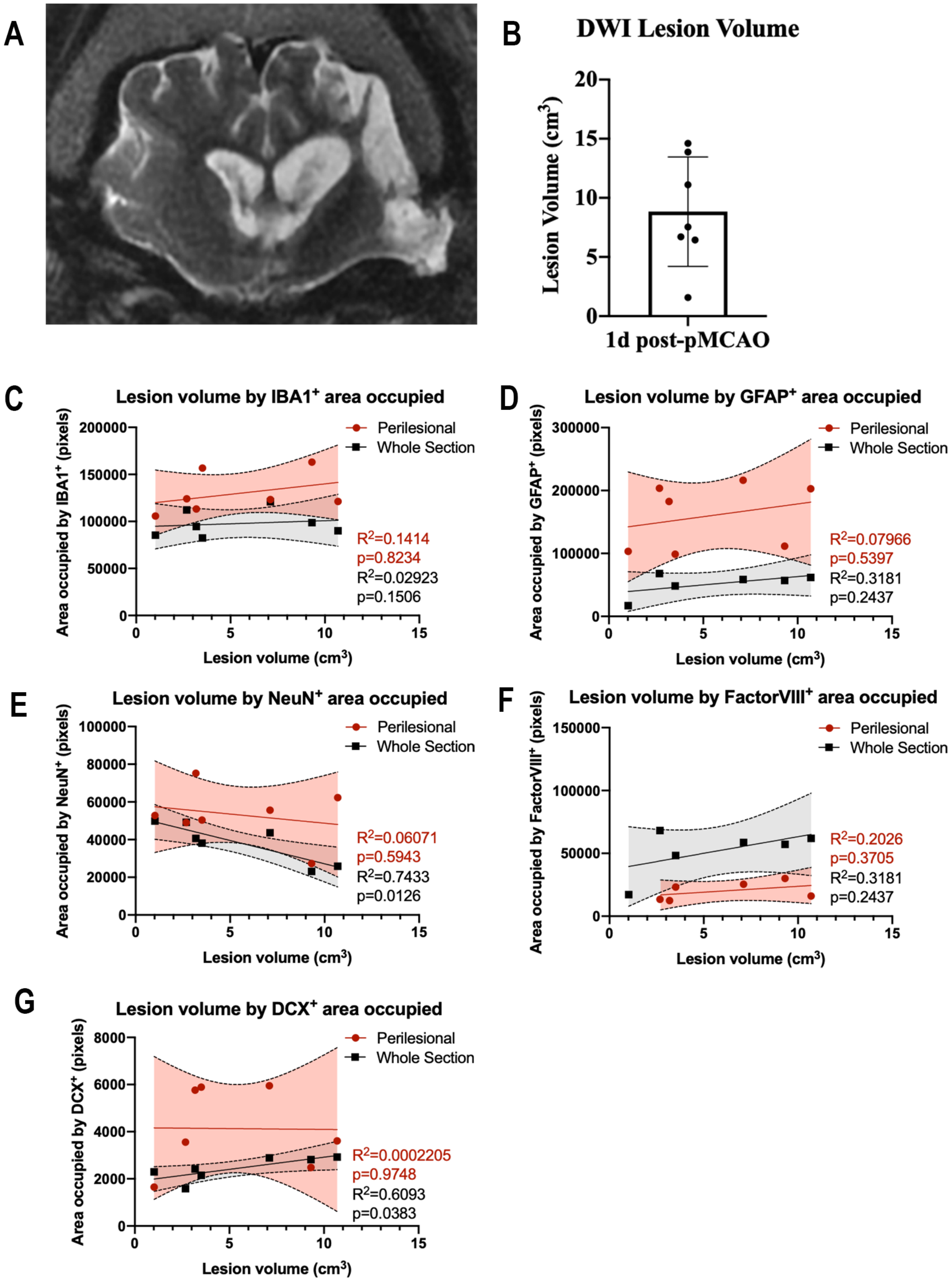

Supplementary Figure 9. Correlations of lesion volume to positive areas of IBA1, GFAP, NeuN, FactorVIII, and DCX. T2W MRI image depicting lesion volume at 1d post-pMCAO by

the hyperintense region in the ipsilateral hemisphere (**A**). DWI corrected lesion volume was measured for all stroked animals and had a mean lesion volume of 5.36 cm^3 with SD of 3.68 cm^3 (**B**). Linear correlations of lesion volume and stained area were evlauated for IBA1 (**C**), GFAP (**D**), NeuN (**E**), FactorVIII (**F**), and DCX (**G**). A significant negative correlation between lesion volume and $NeuN^+$ areas (p=0.0126) was observed in whole slice analysis (**E**). Comparatively, a significant positive correlation between lesion volume and DCX^+ areas (p=0.0383) was observed in whole slice analysis (**G**).

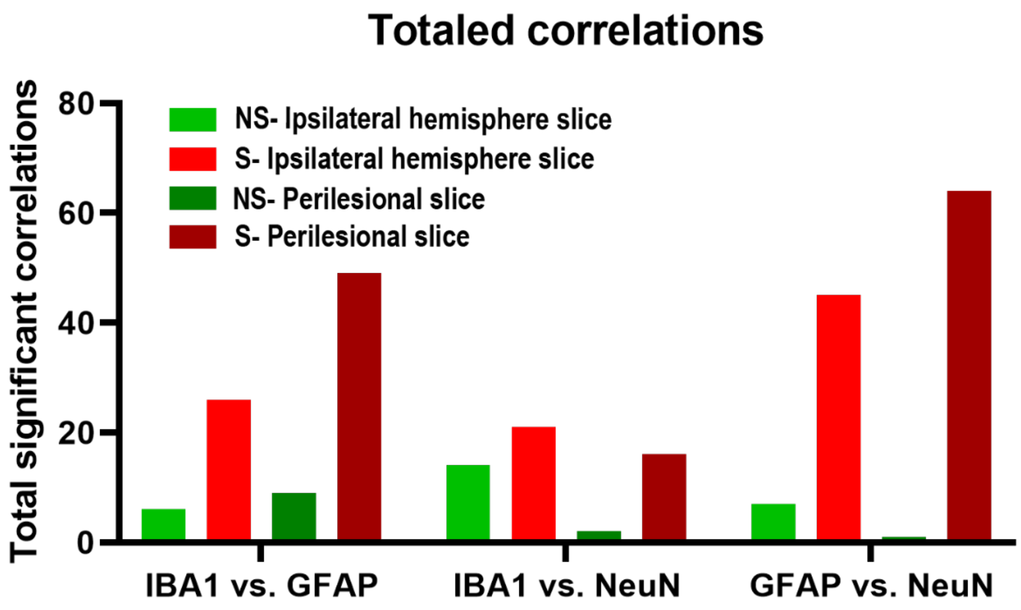

Supplementary Figure 10. Quantified significant correlations between stains and treatment groups. Number of significant correlations for $IBA1^+$, $GFAP^+$, and $NeuN^+$ areas were quantified for each treatment group (NS= green, S= red), and locations (Ipsilateral section = lighter shade, Perilesional area = darker shade). Greatest number of correlations were observed between $IBA1^+$ vs. $GFAP^+$ and $GFAP^+$ vs. $NeuN^+$ in S perilesional areas than any other group. Greatest number

of correlations between $IBA1^{+}$ vs. $NeuN^{+}$ was observed in the ipsilateral hemisphere section of S animals.
